# Microbiome First Approaches to Rescue Public Health and Reduce Human Suffering

**DOI:** 10.3390/biomedicines9111581

**Published:** 2021-10-30

**Authors:** Rodney R. Dietert

**Affiliations:** Department of Microbiology and Immunology, Cornell University, Ithaca, NY 14853, USA; rrd1@cornell.edu

**Keywords:** microbiome, public health, chronic diseases, microimmunosome, eating disorders, substance use disorder, commensals, pathobionts, sensory receptors, developmental programming

## Abstract

The is a sequential article to an initial review suggesting that Microbiome First medical approaches to human health and wellness could both aid the fight against noncommunicable diseases and conditions (NCDs) and help to usher in sustainable healthcare. This current review article specifically focuses on public health programs and initiatives and what has been termed by medical journals as a catastrophic record of recent failures. Included in the review is a discussion of the four priority behavioral modifications (food choices, cessation of two drugs of abuse, and exercise) advocated by the World Health Organization as the way to stop the ongoing NCD epidemic. The lack of public health focus on the majority of cells and genes in the human superorganism, the microbiome, is highlighted as is the “regulatory gap” failure to protect humans, particularly the young, from a series of mass population toxic exposures (e.g., asbestos, trichloroethylene, dioxin, polychlorinated biphenyls, triclosan, bisphenol A and other plasticizers, polyfluorinated compounds, herbicides, food emulsifiers, high fructose corn syrup, certain nanoparticles, endocrine disruptors, and obesogens). The combination of early life toxicity for the microbiome and connected human physiological systems (e.g., immune, neurological), plus a lack of attention to the importance of microbial rebiosis has facilitated rather than suppressed, the NCD epidemic. This review article concludes with a call to place the microbiome first and foremost in public health initiatives as a way to both rescue public health effectiveness and reduce the human suffering connected to comorbid NCDs.

## 1. Introduction

While public health began life with great promise such as the impact of sanitation and clean water [1], it has been on a slippery slope of repeated failures particularly during the 21st century [2,3,4,5,6,7]. This review article illustrates that: (1) lack of recognition of the fundamental nature of humans (superorganisms/holobionts), (2) a failure to include the vast majority of human genes in public health initiatives (i.e., the human microbiomes), (3) failure to account for the role of microbiome dysbiosis in the majority of human deaths (inflammation-driven noncommunicable diseases and conditions (NCDs)) and (4) failure to recognize and/or eliminate NCD-promoting food additives, chemicals, and drugs has completely undermined decades worth of public health programs. This article provides examples of recent public health failures that each impact the battle against the ongoing epidemic of chronic disorders also known as NCDs. 

Importantly, the article suggests a way back to meaningful public health success by undertaking microbiome first approaches to attack the NCD epidemic. Three specific categories of microbiota impacting public health are introduced as examples of microbiome first approaches. The paper also discusses the critical need for public health effectiveness in both education and action if we are to achieve sustainable healthcare. 

It is of note that I am not the only one to call for a complete overhaul of public health to one that embraces the human holobiont and prioritizes microbiome-based health solutions. In their paper “A Framework for Microbiome Science in Public Health,” Wilkinson et al. [8] make a similar appeal. As will be described in the following section, public health initiatives in the past several decades have been nothing short of a catastrophe. This paper argues that the only way forward to meaningful, relevant, and effective public health is: (1) to give our majority microbial copartners their due, and (2) to recognize that the ability of “public” health to actually reduce the prevalence of chronic disease (versus the unfettered growth of NCDs) can only occur when microbes are managed for the greater good.

## 2. Recent Failures of the Public Health Promise

In a 2004 editorial in the medical journal The Lancet titled “The Catastrophic Failures of Public Health” [3], the editors castigated 21st century public health institutions for their misdirected focus. The editorial pointed out that the real public health threat was not the more glamorous pandemics such as SARS or avian influenza. Instead, it was the simpler and less glamorous reality that more people are becoming obese and sedentary, and are “more prone to killer chronic illnesses, such as cardiovascular disease, stroke, cancer, and diabetes.” As proof, the editorial went on to cite CDC statistics showing that human illness and death overwhelmingly occurred because of the NCDs and not the pandemics [3]. The Lancet’s 2004 call for Public Health Institutions to focus on the main causes of human suffering and death went unnoticed at least based on public health outcomes. Enter the shiny new glamorous pandemic, SARS-CoV-2.

Even in the face of the current SARS-CoV-2 pandemic with massive numbers of “public mandates” still failing to resolve the outbreak of infection, global deaths are predominantly caused by NCDs such as obesity and its comorbidities. Seventeen years have passed since The Lancet dressed down Public Health Institutions and little has changed toward reversing the NCD epidemic. Furthermore, the risk of comorbid diseases connected to what are often childhood onset NCDs (e.g., asthma, obesity) presents a staggering health challenge across the life course. For example, a diagnosis of childhood or adult asthma carries with it an elevated risk for at least 36 additional NCDs. For obesity, the prospects of a life course filled with increasing disease are even worse. There are 43 recognized comorbid NCDs linked with obesity despite recent medical and public health efforts [9]. A recent National Health and Nutrition Examination Survey (NHANES) study reported that among seniors in the U.S., the rate of two or more NCDs is a staggering 91.8% [10]. This can only be regarded as a public health failure. We have been staying the course in public health for too long and produced multimorbidity and reduced quality of life as public health outcomes. 

## 3. The Blame Game

In general, public health organizations such as the WHO, CDC, FDA, NIH, and EPA have placed the reason for the NCD epidemic squarely on the poor behavior of the public. If only the public would change their behaviors, the leading cause of global death would disappear. Presumably, we would return to the circumstances where death certificates read “died of natural causes,” and old age would be a blessing rather than being an NCD-ridden, drug-addled existence. However, a look at the very behaviors that organizations like the WHO say will solve the problem are less and less under the control of the individual. In fact, in this opinion article, I will argue that those behaviors are greatly impacted by the human microbiome, and the already-depleted human microbiome is under virtual assault from the actions of the very organizations that should be ensuring its safety. When government-mandated and/or -approved practices are inherently unsafe for the human microbiome, the “fault” in not changing microbiome-determined behaviors is no longer on the individual. 

## 4. Change Is Overdue

At the heart of the public health problem is that public health organizations, academic and governmental bureaucracies have failed to recognize significant new science when it comes to public application. For example, most schools of public health have faculty lines and research centers committed to microbiome research. However, these research initiatives seem to be completely disconnected from actual public health intervention programs. How does that happen? In fact, the same 2004 editorial in The Lancet [3] could be republished today changing only SARS to SARS-CoV-2 and provide the same reality check for those institutions that practice public health. Academic public health programs need to make their new staffing count with microbiome-delivered solutions. We cannot wait a half century or more for a Microbiome-First NCD epidemic solution to bubble up. According to the World Economic Forum—Harvard School of Health report on NCDs [11], we do not have the luxury of even another decade of an unrelenting NCD epidemic.

## 5. The Updated Science–Application Gap: Ancient Personal Responsibility Solutions to Stop NCDs

The epitome of this reality gap in public-health related research and public health policy and initiatives can be seen in a comparison of two current World Health Organization (WHO) public webpages. The WHO appropriately emphasizes that NCDs cause the vast majority of global deaths (71%) [12]. The threat goes beyond that of NCDs killing more humans in every country with each increasing year. As the World Economic Forum and the Harvard School of Public Health pointed out, the world cannot avoid the economic burden of the NCD epidemic. The human and economic toll is unsustainable. With the WHO having a clear vision of the threat, one would assume that this global public health organization is currently mobilizing the latest science to address this most serious human health challenge. That assumption would be incorrect. The WHO currently recommends that “cures” for the half century long NCD epidemic can be found in reducing four risk factors that involve four human behaviors: “tobacco use, physical inactivity, unhealthy diet and the harmful use of alcohol” [13]. 

This is not a new discovery by public health institutions nor is it a new “public health” effort. In fact, virtually the same health proclamations used by the WHO originated from ancestral healers dating back millennia to the time of the “Four Humors”. Simply put, this is not new science as is discussed in the following. Despite centuries if not millennia of similar WHO-like behavioral admonitions, the NCD epidemic emerged during the 20th century and now in the 21st century is unrelenting. For example, the British medical journal published a study linking alcohol consumption and risk of cancer in 1903 [14]. A publication appeared in The Hospital in 1901 describing tobacco use and cardiovascular disease [15]. The sometimes public admonitions about diets are centuries old as detailed by Foxcroft [16]. Finally, recognition of the connection between inactivity and disease including NCDs is not new. Physicians in the ancient Indus Valley, Greece and Rome actually wrote prescriptions for exercise (reviewed in [17]). What is newer is the increased understanding that NCDs and inactivity can be a vicious cycle. NCDs such as gout make health-promoting exercise even more difficult as was noted by Benjamin Franklin [18].

As can be seen in Table 1 [3,4,9,10,13,19,20,21,22,23,24,25,26,27,28,29,30,31,32,33,34,35,36,37,38,39,40,41,42,43,44,45,46,47,48,49,50,51,52,53], the danger in recent public health failures is not simply that time and money was expended in unsuccessfully combatting the NCD epidemic. It is that some of the recommendations actually further erode both the microbiome and human health. Several initiatives were not even health neutral. 

## 6. The Global Burden of Disease Study

In addition to the ancient dogmas perpetuated by the WHO, other promising initiatives have also ignored human microbiome status as a risk factor for NCDs. One example is the massive and ongoing Global Burden of Disease Study centered in Australia. The study includes analyses of 87 risk factors across more than 200 countries [19]. However, none of these risk factors directly pertain to the microbiome. The closest relevant indicator involves breast feeding practices. Many of the risk factors are direct outcomes of microbial dysbiosis (e.g., metabolic profiles) but the majority causative gene pool (microbial) determining relevant metabolic status is not considered. It is worth asking how long this study must go on before the microbiome and NCDs are included. It can be argued that to date, this massive study has been working on the margins of core risk factors for the human superorganism. The factors tracked across more than 200 countries fall more into the category of general umbrella behaviors as well as downstream “effects” of microimmunosome dysbiosis/misregulation rather than core causes of NCDs. The only exception in the study might be the inclusion of breastfeeding behaviors. More human systems biology-relevant parameters based on research of the past decade would be useful. These would focus on direct causes of: (1) improperly seeded, fed and/or damaged human microbiomes, (2) loss of barrier function, (3) pathobiont predominance, (4) changes in bile acid and other metabolism, microbial production of short chain fatty acids and regulatory peptides, and (5) induction of immune inflammatory dysregulation [9].

## 7. NHANES Results Illustrate That Multimorbid NCDs with Polypharmacy Are the New Norms

As was discussed by Dietert [9], a recent NHANES study provided a stunning indicator of the failure to address NCDs among the U.S. aging population [10]. The NHANES surveys are not a new public health initiative. Instead, they are a regularly occurring, comprehensive, and comparative group of indicators that illustrate public health progress or failure among a large sampling of the U.S. population. 

## 8. Failure to Protect Multimorbid NCD-Bearing, Pro-Inflammatory Seniors against the SARS-CoV-2-Induced Cytokine Storm

As discussed in Dietert [21], SARS-CoV-2 showed us the extent to which the ongoing NCD epidemic had not been adequately addressed via preventative and therapeutic medicine. Importantly, it is the additional lack of success in public health that allowed the NCD epidemic to progress. With decades of public health initiatives having failed to halt the multimorbid march of NCDs with aging, it was a double tragedy that the most vulnerable population for death by SARS-CoV-2 (those with multimorbid NDC burdens, a damaged microimmunosome and with misregulated inflammation) were not adequately protected. Because cytokine storms represent a misregulated inflammation response in tissues and because of the proven risk of the geriatric cohort to secondary bacterial pneumonia, the selective targeting of the segment of the population by SARS-CoV-2 was easily predictable. Yet, despite our clear understanding of NCD-related immune dysregulation, the public health response was poor [54,55].

## 9. The National Children’s Study

The National Children’s Study (NCS) was a significant inter-federal agency early life health effort to address the increased environmental vulnerability of children for risk of disease and the nature of developmental programming for both infections and NCDs [26]. It was created by the Children’s Health Act of 2000 and would eventually enroll H.R.4365—106th Congress (1999–2000). As described by the NIH Children’s Health Study Archive site (https://www.nichd.nih.gov/research/supported/NCS; accessed on 26 August 2021) the Pilot study began in 2009 and enrolled 5000 children across 40 U.S. locations. The primary study was designed to have followed 100,000 children. It involved a pregnancy to adulthood study with exposure–outcome indicators. Because of the ideas surrounding critical windows of developmental vulnerability [56], developmental origins of health and disease (DOHaD) [57], and powerful research findings on transgenerational epigenetic programming [58], the NCS held great promise. The original Congressional Act specified such NCDs as autism, juvenile diabetes and other NCDs. After only five years into the program, the NIH Director closed the NCS in 2014. What had begun as the promise of providing key information into the early life programming of NCDs became little more than a very expensive, truncated trial. The death of the NCS led to what has been termed scientific humiliation at a cost of 1.3 billion dollars when then and present Director of the NIH, Francis Collins, killed the program [28]. 

Public Health done correctly with the NCS would have produced highly useful results for protecting prenatal, infant, and childhood periods of vulnerability. Additionally, had the microbiome been included in the plan, the fact that microbiota determine individual risks to food, drugs, and environmental toxicants would have been revealed and might have led to better preventative medicine against NCDs sooner rather than later [9,59,60,61,62]. 

## 10. Public Health Failures among Regulatory Agencies

Two categories of public health regulatory failures are listed in Table 1. The first concerns major misses or “regulatory gaps” in safety evaluation resulting in millions of people across more than one generation being exposed en masse to toxic drugs, chemicals, or food additives. These exposures were later shown to contribute to both system(s) dysfunction and one or more NCDs. The first category illustrates general toxicity misses not necessarily linked to the microbiome. The second category concerns the lack of relevant safety testing specifically involving the microbiome.

Dietert and Dietert [63] discussed the problem with public trust for FDA and USDA stamps of approval when it comes to safety including that for the human microbiomes. However, the issue of effective public health protection by regulatory agencies goes beyond just the tendency to embrace outdated science and cling to decades old, status quo, safety evaluation strategies. The problem is that once the errors in safety testing and human health protection are revealed, massive NCD-promoting exposures have already occurred and offending toxicants may or may not be removed from use by the responsible public health agencies. For example, food emulsifiers are clearly obesogens via their capacity to destroy the keystone gut bacteria, *Akkermansia mucinophila*, compromise the gut barrier, allow pathobionts to gain predominance, and produce underlying immune-inflammatory dysregulation [64,65]. Yet, the FDA has not acted to protect consumers from this pervasive food additive hazard despite the fact that reducing the prevalence of obesity is among the highest public health priorities [66,67]. When the WHO directs people to eat healthier foods to reduce obesity, are they considering elimination of most emulsifier-containing foods or at least replacing emulsifiers with a microbiome friendly alternative?

The public health problems extend beyond food additives to environmental chemicals that can also reach us via the food chain. As a result in the U.S., the EPA, the National Toxicology Program (NTP) and the FDA were all involved in the safety evaluation surrounding major plasticizers such as bisphenol A (BPA). Despite BPA being an endocrine disruptor, a widespread chemical, and having a variety of toxicology red flags that show up over the years, the politics of BPA made elimination of exposure challenging [68]. While BPA has adverse effects on many different tissues and organs (particularly NCDs involving the reproductive system), recent studies suggest that it is an obesogen [69,70]. Pérez-Bermejo et al. [71] recently reviewed the role of BPA in obesity and diabetes and concluded that it can stimulate adipocyte hypertrophy and disrupt glucose metabolism and insulin homoeostasis. Exposures in early life present the greatest risk of BPA promoted obesity. The researchers concluded that endocrine disruptors like BPA likely contributed to the increased prevalence of obesity. They also note that while some countries have taken steps to limit exposure of their population to BPA, there is still a lack of international agreement that would globally ban BPA. Finally, BPA has been reported to play a pathogenic role in Crohn’s Disease working though the microimmunosome to increase bacterial translocation and increase systemic inflammation [72]. 

If the WHO wants to reduce NCDs including obesity, getting rid of obesogenic emulsifiers and endocrine disrupting plasticizers would be a great place to start. It is not the public’s fault when good choices in diet, exercise, attempts to reduce tobacco and alcohol use are undermined by massive multigenerational exposures to hidden products that damage the microimmunosome and promote obesity as well as other NCDs. Modifying personal behavior to reduce the risks of NCDs is only useful if microimmunosome damaging drugs, chemicals, and food additives are not embedded within every household food as part of everyday life. Microbiome First oriented physicians, nutritionists and other health practitioners could restore the human microbiome, but inadequate public health actions would erode it yet again on a daily basis. 

## 11. The Final Group of Problematic Public Health-Related Activities

The 2004 medical journal alarm on Public Health (Table 1) and the lack of effectiveness when it comes to NCDs was previously discussed at the beginning of this article. The Lancet article was not a new public health project. Rather, it provided an important timestamp for the ongoing effort of public health institutions to bring the NCD epidemic to an end. The problem is that today’s approach by institutions like the WHO seems to be largely the same 17 years after being called out by The Lancet. It seems clear that a sea change is in order rather than annual tweaking of the same approaches that got us into a healthcare-threatening epidemic. 

One of the great public health promises at the end of the 20th century and the beginning of this century was The Human Genome Project [73,74]. Mapping and analysis of the complete human genome was touted as providing the wherewithal to cure most if not all NCDs. The project did map the human genome, but the results were underwhelming both in terms of number of chromosomal genes and the impact of that information on human health [75]. The silver lining in this failure was that it paved the way for The Human Microbiome Project and the 100-fold-plus number of human microbial vs. chromosomal genes that would be tallied. It should be clear now that the promised cures for NCDs reside minimally among our mammalian chromosomes and to a much greater extent belong to our microbial copartners. 

In 1976 a rare diagnosis of swine flu on the Ft. Dix military base in NJ led to what in hindsight was an overreach and overreaction of U.S. national public health. The feared pandemic never emerged, but a rushed vaccine that was administered nationally by the government did. The risk–benefit was poor with Guillain–Barre Syndrome among the adverse outcomes [29,30].

As shown in Table 1, a local public health-related initiative in Flint, MI led to a change in water supplies producing an almost unthinkable outcome [76]. It also showed that the breakdown of deliverable public health was not simply at one level of administration. 

Ironically, during the later 1990s to early 2000s, federal agency grant programs, like the external funding program of the EPA, went through a period where research proposals to examine toxicity of environmental chemicals specifically excluded the heavy metal lead. Proposals on any other environmental chemicals were allowed. Because the author had been researching lead over this period, these exclusions brought the lab’s lead immunotoxicity research to an end. The logic was that we knew everything we needed to know about lead or at least enough to know that we must avoid the exposure of children to lead at all costs. Yet, decades later in Flint, MI, public health protection failed us as has been noted by some of the most prominent environmental health researchers [4,50].

The examples of public health failures listed in Table 1 show that rather than solving the most lethal health crises of the past half century (NCDs), an assault on the human superorganism has been permitted to continue where large-scale public exposures are permitted to occur before the actual, relevant risk–benefit is known and made public.

Mass exposures to bisphenol A should not have happened and daily exposure to glyphosate, food emulsifiers, and microbiome-damaging drugs should not be occurring. Dedication to the protection and nurturing of the human microbiome is probably the single most effective tool that public health initiatives could embrace to end the epidemic of lethal human diseases, the painful suffering of populations with multimorbid NCDs, and the reduced quality of life over much of the life course.

Finally, the SARS-CoV-2 pandemic and the public health push for global mass vaccinations led to a remarkable seeming amnesia on a fundamental basis of immunology: the development of natural immunity and protection of the host via heterologous adaptive immune responses. The responses are tailored to the protection against the pathogen since they engage the millennia-honed combination of innate and adaptive immune processes that were effective against the category of pathogens across centuries and are characterized by both specificity and memory. When public health challenges arise, it is definitely not the time to forget fundamental natural disease resistance processes established over decades and funded by the very same institutions responding to current health crises. This would be a prescription for continuing the record of poor public health initiative outcomes reflected in Table 1.

## 12. Transforming Public Health for Impactful Successes against the NCD Epidemic

This review provides three inter-related categories of microbiome-based defense against the ongoing NCD epidemic that have the ability to transform the recent string of public health failures/tragedies into meaningful progress in the fight against the NCD epidemic. This is not intended to be an exhaustive list of microbiome-based approaches to combat NCDs, but are simply examples illustrating how public health can and should change its focus to be compatible with the biological reality of humans (i.e., as superorganisms/holobionts).

The first category focuses on the integrity of the microimmunosome and, in particular, barrier function (e.g., skin, gut, airways, urogenital tract). There is an emerging concept that barrier protection should be a prime directive in the battle against NCDs such as the allergic triad [77]. Consider when and where compromising barrier integrity is a healthful change, and the problems can be put into perspective. It is never a good thing when bacteria and/or bacterial toxins translocate to part of the body where those bacteria do not belong. That is one of the fastest routes to disease and sometimes sepsis. 

Starting with the skin, the largest organ in the body, there are commensal microbes that both regulate the health of the skin barrier and provide colonization resistance against pathogenic bacteria and viruses. Skin commensals influence cutaneous immune cells affecting the balance of immune inflammation and wound repair as well as innate immunity against vaccine viruses [78]. Clearly, skin commensals offer a manageable and useful strategy to ensure natural protection against pathogens entering via the skin as well as improper immune inflammatory responses. Recent studies on skin microbes suggest that strains matter. It is the actual collection of genes with *Staphylococcus epidermidis* (Staph E) that determine the extent to which it affords potent antimicrobial protection. A recent study suggested that transplantation of Staph E and *Staphylococcus hominis* works in animals, and that peptides from these commensals kill *Staphylococcus aureus* (Staph A) [79]. Finally, a recent meta-analysis suggests that probiotics can be effective in preventing childhood atopic dermatitis providing a new avenue for reducing the risk of NCDs [80]. In double blind, placebo-controlled studies, certain probiotic strains were found to be beneficial in the treatment of childhood atopic dermatitis [81]. A full range of skin transplantation competitive exclusion strategies was recently reviewed by Callewaert et al. [82].

In mucosal tissues where the barrier is protected against pathobionts by active regulation of the mucin layers, there are key microbial biomarkers that affect both barrier status and risk of specific NCDs (e.g., obesity/metabolic syndrome). In the gut *Akkermansia mucinophila* is known as a Keystone species because of its critical role in mucin regulation and protection of the gut barrier. It is one of a small number of bacteria that can perform this critical function. Risk of obesity and metabolic syndrome in general is inversely correlated with the levels of this bacterium [83]. As a result, this bacterium has been labeled as the “sentinel of the gut” [84]. Recently identified pilli-like outer layer proteins on the bacterium are important in both immune regulation and effective barrier function [85].

Any public health organization worried about the prevalence of obesity and metabolic syndrome related NCDs, should be worried about measuring and monitoring *Akkermansia mucinophila.* Eating healthier diets will mean little if other factors are destroying a person’s gut barrier protecting bacteria. There are specific dietary factors that serve as prebiotics for *Akkermansia* [86]. However, if the WHO is not focused on educating the public about the microimmunosome (the microbiome, a healthy barrier, and the underlying immune system) and specific prebiotics to aid the gut lining, then simply pushing the public toward what is perceived as “a healthier diet” may not have the intended effect.

In fact, it appears that *Akkermansia mucinophila* can also be useful as a probiotic for improving the safety of drugs with harmful side effects in the gut. One case is for the antipsychotic drug, olanzapine, which causes disrupted glucose homeostasis. In lab animal studies, probiotic *Akkermansia mucinophila* strains eliminated this metabolic syndrome inducing side effect [87].

In the airways, epithelial barrier status is critical in the risk of a wide array of airway diseases [88]. Additionally, early microbiota–immune interactions in the airways determine the course of airway mucosal immunity and risk of airway disease [89]. One of the considerations for the airway microimmunosome is whether the pathobiont Staph A is carried in the infant nasal passages. As recently reviewed by Coleman et al. [90], the carriage of Staph A presents an increased risk for asthma (and other allergic conditions). This Gram-positive pathobiont has toxins and enzymes that can serve as allergens and sensitizing agents for the mucosal immune system [91]. Additionally, it can stimulate Th2 inflammatory cytokine production by epithelial cells that can result in barrier damage. The infant epithelial lining is particularly susceptible to pathobiont-associated damage because there is decreased production of epithelial-derived antimicrobials in early life [92]. Because antibiotics can often lead to only a short-term benefit with longer term complications as a probable outcome, effective colonization resistance against Staph A in the infant airways is the best benefit–risk strategy [93,94]. As was discussed for the skin, Staph E is a key bacterium in nasal maturation and natural defenses against pathobionts like Staph A [95]. However, this can only happen when ecologically managing microbes is a medical and public health priority. 

## 13. The WHO and Its Four Modifiable Behaviors to Defeat NCDs

In attempting to halt the ongoing NCD epidemic and eradicate NCDs, the WHO has focused on four modifiable behaviors. Rather than a true public health strategy based on recognition of the human holobiont and microbiome-exerted control over much of human behavior, the WHO has defaulted to a single-species educational program designed to admonish the public on their own shortcomings. Essentially, the WHO’s solution to NCDs is that if only the public changed what they do, NCDs would go away. However, as this review will show, the very behaviors the WHO believes are readily changed in each individual are in many cases only changed if and when the dysbiotic microbiome is rebiosed. 

Public Health programs will never return to their early, glory day successes until and unless they embrace and apply Microbiome First approaches and proactively help people to usefully manage their copartner microbes. NCDs are not the fault of the public when the most common food, drug and environmental chemical exposures damage the human microbiome. Rather NCDs are more the fault of woeful public health regulatory activity that fails to protect the human (and other) microbiomes.

The following sections illustrate how the microbiota impact and control the WHO’s NCD-relevant human behaviors. Because the WHO indicate that the four most significant risk factors for NCDs involve personal behavior, the WHO’s direction for individuals to change these behaviors (in the absence of holistic public health support for these changes) is a form of mandate with similarities to what has been seen during the SARS-CoV-2 pandemic. 

## 14. WHO Behavioral Modification #1: Eat a Healthy Diet (in Spite of the Microbiota-Driven Sense Control)

We experience life much through our senses. However, what is becoming clear is that sense-driven life experiences are significantly affected by our microbiome. If you want to get more out of life and have richer experiences, rebalancing the microbiome is the first place to start. We can go back to the WHO’s approach to solving the NCD epidemic in general and the obesity epidemic specifically by admonishing people for eating a poor diet and telling them to eat healthier. The public messaging belies a lack of practical consideration on how one overcomes microbial control of the body’s taste sensitivities, food choices, and eating behaviors to facilitate a holistically compatible shift in diet.

We now know that our partner microbes have a myriad of ways to affect virtually every aspect of our diet, food consumption, food preferences, and nutrient extraction from the foods we do eat. One of the key starting points for our relationship with food is taste (as well as smell). Our oral and gastrointestinal microbiota are essentially the miners of the nutrients we receive from our food. What we get from food depends upon what we eat but also on what the microbes do as per extraction and metabolism of the food. Table 2 [96,97,98,99,100,101,102,103,104,105,106,107,108,109,110,111,112] illustrates examples of research into the relationship between taste, food choices, and eating. Other factors such as smell/odor detection, satiety, and addictions follow in subsequent tables. Importantly, the following tables also demonstrate the key role of our microbiota in determining both threshold levels for taste and smell behavioral reactions to those cues. It affects not just our food preferences and eating behavior, but also our appetite and satiation. In the end microbiome dysbiosis plays a central role in eating disorders and can drive unhealthy eating both consciously and unconsciously. The take home message across the studies is that in order for the WHO’s instruction to eat healthier to produce a truly successful outcome, the individual’s microbiota need to be congruent with the taste, smell and energy sources associated with a healthy diet. 

For the purpose of considering the inter-relationships between regulation of the senses, food behavior, and risk of NCDs, I will use obesity/metabolic syndrome as a specific example. Obesity is one of the key drivers of the ongoing NCD epidemic and is one of the first NCDs to arise during childhood. Additionally, as reported by Dietert [9], the cohort diagnosed with obesity is at a greater risk than the general population for at least 43 comorbid NCDs across the lifespan. As a result of these comorbidities, the metabolic syndrome complex which includes obesity is one of the most insidious plagues on modern humans and a huge contributor to both premature death and reduced quality of life.

Not surprisingly, the public health solutions for the obesity epidemic must overcome two massive roadblocks: (1) rampant microbiome dysbiosis that locks in destructive eating behaviors and (2) the inexplicable presence of obesogens permeating both our environment and most of our food. Rather than simply telling people to eat a healthier diet and then being mystified by the poor outcomes among the NCD-at-risk and NCD-burdened populations, public health organizations should be showing people how to align their microbiome with eating a healthier diet. Secondly, public health organizations should get obesogens out of the environment and food chain. They should never have been there in the first place, and they certainly should not be there now. Microbiome rebiosis can only work if the almost constant exposure to obesogens is eliminated. 

### 14.1. Taste

Do gut microbes taste? [96] (See Table 2). Leung and Corvasa [96] address this in their recent review. There are five tastes that humans can both detect and evaluate for quality: sweet, bitter, salty, sour, and savory. It is also possible we can distinguish fats (e.g., linoleic acid). The tongue is a perfect design for biofilm formation. Depending upon the thickness of a bacterial biofilm on the tongue, taste receptors can be blocked from engaging specific foods. 

Gut microbiota control taste through three inter-related processes. First, they can directly interact with the barrier between food and taste receptors, screening and/or blocking access to your taste receptors. Blocking the taste receptors through biofilm formation can have consequences for food intake and eventually health. Secondly, our microbes can affect taste through hormone interactions.

Finally, microbiota affect taste via the immune system. Taste receptor bearing cells have to turn over and inflammatory processes resulting from loss of barrier function and/or colonization resistance can allow lipopolysaccharide (LPS) to activate innate immune cells increasing proinflammatory cytokine concentrations. As these spread, both taste cell receptor expression and taste thresholds change [96]. This is another reason why barrier integrity is crucial. Taste buds can have a delayed renewal and a shorter lifespan when microbiome–immune interactions go wrong. Of course, this is the steady state physiology when patients carry NCDs. Microbiome dysbiosis-induced inflammation essentially kills taste buds. The microimmunosome must be engaged first if you want people to readily shift to a healthier diet. 

Several recent studies focused on very specific microbiota regions and also very specific taste biases. The importance of the oral microbiome for taste and flavor to dental health was recently described by Ellender and Moynihan [97] with an emphasis placed on the life course/aging impact of flavor biases. 

Jurczak et al. [98] conducted an important study of preschool children (2–6 years of age) comparing sugar threshold perceptions, amount of sugar consumed, dental caries, and culture based oral cavity bacteria and yeast. The results showed that the presence of *Streptococcus mutans* was associated with a poor sugar detection profile (high threshold concentrations of sucrose were required before it could be perceived), higher sugar consumption, and higher prevalence of dental caries. This is one example of a key bacterial marker and sweet detection profile that drives specific food consumption and resulting oral pathology.

A second study of oral health was a crowdsourced sampling population study conducted via the Denver Museum of Nature & Science [99]. One interesting observation was that an oral pathobiont, *Treponema*, was detected most often in adults with dental problems and in obese youth. Li et al. [100] discuss the tongue microbiome, its effect on taste receptors and the routes through which tongue microbiota dysbiosis can directly and indirectly promote obesity, diabetes, and cardiovascular disease.

Dysbiotic microbiota can drive taste bias that promotes a vicious cycle of eating foods that both maintain the specific dysbiosis and cause inflammatory-driven disease. Three studies illustrate these cycles for microbial control of sugar craving and fat craving. Esberg et al. [101] connect specific communities of oral microbiota with elevated prevalence of dental caries and high sugar food intake. Two studies from Besnard et al. [102,103] dealt with oral microbial regulation of fat taste sensitivity as relates to obesity and diabetes. In the first study by Besnard et al. [102], the microbial composition of the mouth’s gustatory circumvallate papillae (CVPs) was analyzed and compared among obese adult men as it related to low vs. high sensitivity of fat taste perception. The decreased fatty taste perception in low-lipid tasters was associated with elevated levels of *Bacteroides* genus and *Clostridium*_XIV bacteria and a decreased level of *Lactobacillus* bacteria compared with the corresponding microbial composition in the high lipid taste perception group. Based on an analysis of metabolic pathways, the investigator hypothesized that prevalence of methanogenesis pathways may be directly, positively correlated with fatty taste sensitivity. Note that fatty taste levels were independent of adiposity itself. Hence, the two taste sensitivity groups are likely to represent two distinct subtypes of obese individuals. In their second parallel study, Besnard et al. [103] examined diabetic patients for microbiota signatures in their CVPs in relationship to their fatty taste perceptions. Microbiota have been shown to affect sugar–fat perception balances via sensitivity-resistance to glucagon-like peptide-1 (GLP-1). The two takeaway findings were that insulin resistance itself does not appear to control fatty acid sensitivity perception. Instead, that is related to the microbiota. Additionally, taking some drugs (e.g., metformin and statins) was found to affect fatty acid sensitivity [103].

In an important proof of concept study in rats, Pocheron et al. [108] performed cecocolonic and cecal content microbiota transfer experiments between selectively bred, obese-prone (OP)/obese-resistant (OR) Sprague–Dawley dams into Fischer F344 recipient pups from birth to 15 days of age. F344 sham inoculated pups were also evaluated. The inoculums contained different microbiota compositions. The different donor microbiota that were transferred programmed F344 eating behaviors even into adulthood. This was regardless of the duration of persistence of donor microbiota profiles in the recipient gut. The author concluded that neonatal microbiota profiles could program adult eating behaviors. This would be consistent with the Developmental Origins of Health and Disease (DOHaD) evidence [113] while demonstrating that the microbes themselves can program neonates.

In the Bernard et al., 2019 study [109] from Table 2, diet-induced obese mice were given an inulin-type fructan prebiotic supplement for 12 weeks. The supplementation partially corrected diet induced profiles. Importantly, it shifted both the cecal microbiota profiles toward reduced loss of barrier integrity and inflammation while simultaneously improving orosensory perception of sweet taste and associated behavioral changes. This study emphasizes the utility of using both diet/prebiotics and microbiome rebiosis to naturally produce healthier shifts in eating behavior. The question is, when will such holistically supportive, microbially-based solutions become core to public health organization initiatives (e.g., WHO)?

Research studies in Table 2 demonstrate that one of the most effective ways to change diet is to change the microbiota. Heys et al. [110] illustrated a proof-of-concept study that changing microbiota can change dietary preferences. If the dietary preferences easily sync with a healthier diet, then changing to a healthier diet will not be opposed by the body’s physiology-regulating microbiota. A study by Leitão-Gonçalves et al. [111] also supported the role of gut microbiota in controlling feeding behavior. By shifting the gut’s microbial composition such that it promotes a healthier feeding behavior, one is more likely to succeed with a desired dietary change. This superorganism-based, holistic strategy would facilitate the desire of the World Health Organization that people simply eat a healthier diet (even if it is counter to their dysbiotic microbiome). A gut-microbiota to vagus nerve to brain pathway that can bypass taste receptor status is discussed in the addiction-withdrawal section.

A final point is the effect of high fructose corn syrup specifically on the human microbiome. In a study by Beisner et al. [114], researchers found that different formulation of foods containing fructose resulted in different gut microbiome compositions and metabolism. High fructose corn syrup supplemented foods resulted in the destruction of beneficial butyrate producing gut bacteria and problematic microbial metabolism of host lipids. In contrast, naturally occurring fructose as part of a fruit-based diet appeared to produce an opposite beneficial effect [114].

### 14.2. Smell

Smell is one of the senses that is important in the choices we make in life. Our entire engagement with objects we encounter can be driven by odor detection and both conscious decisions (e.g., moving away from a skunk encounter) as well as unconscious decisions such as selecting one food within a buffet line over another. It draws us to things or away from things based on how we perceive odors. This includes our food as shown in Table 3 [115,116,117,118].

As with taste there are thresholds of odor detection, and there is the nature of the scent itself. One of the interesting tests concerns the tropical fruit, durian. The odor is so repulsive for some that the fruit is inedible. Others savor the flavor so much that the odor is of little concern. The fruit itself has a high alcohol content and can become an obsession for the alcohol-addicted.

Morquecho-Campos et al. [119] recently reviewed the relationship between food odor, congruent appetite, and food preferences. In particular the authors point out that we live in an obesogenic environment. Food groups with similar chemistry and odors can lead to what is termed sensory specific appetite (SSA). 

Nardon et al. [115] found evidence that microbiota can affect the first step in odor detection at the level of the olfactory epithelium. Studies in three different groups of mice examined the effects of microbiota on nasal epithelium and odor detection and preferences in three different groups of mice using physiological, biochemical, behavioral, and microbiological analyses. The investigators used eleven odorants in the evaluation. They found that olfactory preferences were dependent upon two factors: (1) the identity of the odorant and (2) the microbiota profile. Remarkably, microbiota appeared to be capable of differentially activating the olfactory epithelium. Both enzyme production from the epithelial transduction system and electroolfactogram (EOG) signals depended upon the specific microbiota profile. 

Some evidence exists on the mechanisms through which microbiota can regulate olfactory epithelium gene expression. Using two different animal models (mice and zebra fish), Casadei et al. [118] found that nasal microbiota control transcription programs in the host though differential production and activity of specific transcriptional factors. RE1 silencing transcription factor (REST) is a zinc finger transcriptional factor that is regulated by microbiota. In turn, REST affects the gene expression of many neurological system genes by binding to promoter regions. This affects not only olfactory function but also differentiation within sensory organs. 

In a human trial (Table 3), Koskinen et al. [117] found that specific groups of nasal microbiota affected different combinations of olfactory performance/capabilities. These investigators measured threshold detection levels, the capacity to discriminate among odors and the capacity to identify odors. They found significant associations for the presence of specific microbiota groups with odor performance. 

### 14.3. Satiety

While microbiota play a critical role in regulating taste and smell particularly as it pertains to food and food components, the role of microbiota in satiety is a third point in the pyramid of controlling diet and diet-related health. Satiety describes the sense of being full and satisfied as per appetite. The opposite of satiety is intense hunger that can lead to binging on food. It is not just food quality but also food quantity that affects the risk of NCDs [120]. 

Appropriate regulation of satiety is one of the important factors that connects diet, eating behaviors (e.g., meal size), and health. Table 4 [121,122,123,124,125,126,127,128] includes eight examples of recent studies and reviews on microbiota and the regulation of satiety. 

In a recent review, Rautman and de la Serre [121] describe the multilevel influences of gut microbiota on a variety of mechanisms (e.g., both peripheral and central) determining satiety. Evidence suggests that the composition of gut bacteria affects gut–brain communication such as peptide signaling via the vagus nerve. Another level through which microbiota composition controls satiety is by the regulation of both peptide expression and peptide release by endocrine cells within the gastrointestinal system (i.e., termed enteroendocrine cells). Among the peptides affected by microbial regulation are: cholecystokinin, glucagon-like peptide-1, and peptide YY. Beyond appetite-regulating peptide production, microbiota can affect the other end of the regulatory pathway: vagal afferent sensitivity to gut-originating satiety signals [121]. By affecting gut barrier integrity and inflammation, gut microbiota composition affects not only vagal afferent signaling but also the structural integrity of the gut–brain axis (via reduction of VAN numbers and c-fiber withdrawal). 

Central intake mechanisms are also affected by microbiota. As described by Rautmann and de la Serre [121], bacterial inflammatory-inducing products can alter not only sensitivity to CCK and leptin but also cause a neuroinflammation-induced loss of function in pivotal brain regions: the nucleus of the solitary tract (NTS) and the hypothalamus. For example, certain species of bacteria appear to interfere with leptin sensitivity in the hypothalmus [121]. Installation of as few as a single probiotic bacterial species (*Lactobacillus rhamnosus* GG) was found to restore leptin sensitivity in diet-induced obese mice [127]. A final central intake mechanism regulated by microbiota composition is the collection of reward pathways [121]. 

This is particularly relevant to hedonic perception. Microbiota control of the reward pathway will be discussed later in relation to the WHO’s mandate-like public health directive that in order to stop NCDs the public should cease addiction behavior. It should be noted that food addiction behaviors are an important part of addiction cycles, are an impediment to the WHO’s instruction to eat a healthier diet and are significantly affected by microbiome status. Yet remarkably, the WHO’s solutions to the NCD epidemic fail to constructively address this relationship, to inform the public of this relationship, and to provide the public with strategies for breaking the cycle of microbiome-regulated food addiction. 

On satiety, Table 4 provides additional information on the regulation of satiety by specific gut microbiota. Three very recent reviews are included [122,123,124]. Two of the studies focused on obese adult humans while two studies were mouse preclinical studies that investigated microbiota associated with satiety hormone induction. 

## 15. WHO Behavior Modifications #2 and #3: Consume Less Alcohol and Stop Using Tobacco (in Spite of the Microbiome’s Role in Addiction and Withdrawal)

The WHO indicated that priority NCD risk reduction requires both the consumption of less alcohol and cessation of tobacco use. As with the other mandates from the WHO, these are not new health-promoting suggestions. The problem is that they are behavioral modifications that are particularly challenging for individuals to accomplish. The reason is that tens of millions of individuals suffer with substance use disorder (SUD). Such problematic changes are based in part on the addiction related aspects of food, alcohol, and tobacco use.

The problem is that these WHO behavioral modifications are presented as if it is as simple as deciding which fruit to pick up at a grocery store and ensure that the day’s shopping does not include alcohol or tobacco products. However, the instruction to make what seem like simple changes ignores: (1) SUD, (2) the highly addictive nature of these behaviors for many individuals, and (3) the long recognized potential addiction-withdrawal challenges connected to following these recommendations. 

Recent research has shown that the microbiome plays a major role specifically in SUD [129] and in our superorganism body’s reward systems in general. In fact, the gut microbiome uses metabolomic, immune, neurological, and epigenetic mechanisms to control the presence of SUD. If the microbiome is dysbiotic as occurs with microbiota-damaging environmental, pharmaceutical, and/or life course experiences, it may be difficult to nearly impossible to discard food or drug dependencies. Additionally, attempted withdrawal from addiction can be painful and as has been previously reviewed [130], pain is highly controlled by microbiome status. Hence, there are two very good reasons to be “managing microbes” first and foremost. The microbiome can help to minimize the risk of addiction, and/or it can help to increase the likelihood of successful, minimally traumatizing withdrawal. In fact if one wants to modify any addiction, starting with the microbiome is great place to shift the reward–addiction chemical cycle. 

In their recent review, Russell et al. [129] discussed the host genetic factors that are involved in SUD and then further detailed how gut microbiome status can either lock in the disorder or facilitate withdrawal and the likelihood of successful future abstinence. Among the substances reviewed under SUD are: alcohol, cocaine, opioids, nicotine, and cannabis. Similarly, Forouzan et al. [131] reviewed studies concluding that gut–brain axis regulation by microbiota regulate psychostimulant abuse disorders and control the negative affects so important in the potential for relapse. In a similar vein, O’Sullivan and Schwaber [132] concluded that gut microbiota influence visceral–emotional hubs and the anti-reward pathway that is common in alcohol and opioid withdrawal avoidance.

The Russell et al. [129] review article also integrated the information from two prior rodent studies looking at the transplantability of alcohol depressive withdrawal symptoms. In the lab animal study, Xiao et al. [133] performed fecal microbiota transplantation from two-week alcohol-exposed mice into healthy recipients. Donor bacteria took up residence in the recipients, and these animals displayed both depressive behavior and rapid alcohol withdrawal anxiety. Zhao et al. [134] extended this work by transplanting fecal gut microbiota from alcoholic patients into antibiotic-treated C57BL/6J mice. There was colonization by the transplanted microbiota and the recipient mice exhibited symptoms of alcohol dependency. These results suggest that the gut microbiome can carry sufficient metabologenomic information to produce alcohol dependency. Furthermore, other researchers have suggested that psychobiotics (probiotic bacteria affecting brain function and neurochemical balance) could represent a useful therapeutic strategy for alcohol use disorder [135].

The microbiome can impact virtually any addiction where the microbiota can: (1) metabolize the addictive food, drug or chemical, or (2) affect the brain chemistry connected to the behavior. Lucern et al. [136] recently examined the contributions of the gut microbiome–peripheral immune–central nervous system interactions in determining precisely who is likely to develop SUD. 

The concept that the microbiota–drug interaction helps to lock in addiction is supported by Freedman et al. [137]. They found that patients receiving opioids in combination with antibiotics were less likely to become drug addicted upon hospital discharge. Presumably, the antibiotics disrupt the opportunity for a SUD profile to develop in the gut microbiome. Of course, rebiosis of the microbiome after antibiotic treatment is needed for two reasons: (1) to avoid loss to colonization resistance and potential elevated risk of NCDs and (2) to buffer against future SUD.

Evidence suggests that dysbiotic gut microbiota may carry the predisposition for alcohol abuse. Esquer et al. [138] bred generations of alcohol abusing rats and found that antibiotic treatment of rat pups prior to alcohol availability could significantly break the cycle of alcohol abuse. This was further enhanced by the oral administration of a probiotic, *Lactobacillus rhamnosus* GG. The investigators attributed the microbiota-controlled changes to reductions in both an alcohol-related proinflammatory state and sweet taste perceptions with the vagus nerve being key in communication to the brain. 

For humans, Carbia et al. [139] presented an integrative model for alcohol misuse including the gut microbiome–immune system–brain axis. They cited a consistency across species in which *Clostridiales*, *Ruminococcaceae,* and *Lachnospiraceae* are elevated in association with addictive behavior. Within their model, the microbiota in conjunction with dysbiosis-promoted inflammation are key elements of alcohol misuse and addiction. The adolescent period was identified as a window of particular vulnerability for this behavioral programming. In a related paper, García-Cabrerizo et al. [140] argued that because gut microbiota are critical in control of the SUD-associated reward system for drugs like alcohol, psychostimulants, opioids, and cannabinoids, they should be therapeutic targets for reversing SUD. The pathways leading from the gut microbiota to the brain can travel through the vagus nerve, the immune system, the HPA axis, bacterial metabolites, and enteroendocrine cells [140].

Evidence suggests that rebiosis of a dysbiotic gut microbiome can reduce the risk of SUD, aid withdrawal, and reduce the risk of relapse. For example, Novelle [141] concluded that food addiction has many similarities to SUD and that food addiction can be caused by a dysbiotic microbiome. Agustí et al. [142] in a study in rats found that the probiotic *Bacteroides uniformis* CECT 7771 modifies the brain reward response in such a way as to positively impact binge eating disorder. In a mouse study, Thomaz et al. [143] found that withdrawal of morphine-addicted mice from the drug was aided by manipulation of the gut microbiome. 

The tobacco smoking–nicotine cycle has been shown to be intimately connected to microbiome status [144]. For example, a recent study established that there is specific microbiome-based signature in human blood that identifies and distinguishes former and current smokers [145]. Other research groups found that former and current cigarette smokers could be distinguished from never smokers based on fecal microbiota [146]. Additionally, it was shown that individuals with substance use disorders including nicotine addiction can be distinguished from healthy (non-addicted) controls by their oral microbiome [147]. In another study both smokers and users of smokeless tobacco had oral microbiomes that were distinct from those of healthy controls [148]. The microbiome appears to be particularly sensitive to cigarette smoke. In an experiment in mice using third hand exposure (via a cloth exposed to cigarette smoke), mice exhibited differential age dependent effects. The early postnatal period was the most sensitive resulting in not only significant long-term differences in microbiome composition but also important microbial metabolic changes. For example, third hand-exposed mice were significantly elevated in degradation pathways that regulate glycolysis and pyruvate decarboxylation and decreased in coenzyme A biosynthesis and pyrimidine deoxyribonucleoside salvage pathways [149]. Similar effects on the microbiome of children were reported following third hand smoke exposure [150]. 

Because there is a major shift in human gut microbiome composition with smoking cessation, it has been suggested that the appetite–food consumption–weight gain side effect of smoking cessation is likely to be microbiota driven [151]. The likelihood of weight gain upon smoking cessation is an ongoing impediment to smoking cessation [152].

Finally, a study in rats by Simpson et al. [153] provided additional support for microbiome status to be considered first and foremost when it comes to addiction-withdrawal issues. The researchers found that depletion of the gut microbiome caused recruitment/expansion of the same neuronal ensembles across several regions that are involved in both the intoxication to and the withdrawal from oxycodone. The investigators stressed that microbiome status and metabolism are critical when considering responses to substances of abuse including alcohol and nicotine. If the WHO expects to turn their prolonged failure to stop the NCD epidemic into a success, they will have to work through and with the human microbiome.

## 16. WHO’s Behavioral Modification #4: Stop the Inactive Lifestyle and Exercise More (in Spite the Inherent Nature of NCDs)

This WHO behavioral modification initiative to increase exercise is a good general adjunct to support health. Maintaining fitness during aging is useful as part of an overall health program [154]. In specific examples, dancing has been shown to be neuroprotective with aging because it is beneficial in terms of neuroplasticity [155]. Dancing can improve motor impairments, non-motor skills, and quality of life in both Parkinson’s disease patients [156] and those with Alzheimer’s disease [157]. Exercise can reduce cardiovascular risk and improve quality of life in cardiovascular patients [158]. It can also improve the disease management of adolescent obesity [159]. These are all very useful life course interventions.

However, the reliance on exercise to prevent and cure NCDs and thereby stop the NCD epidemic is a questionable approach. While exercise is useful, it is important to recognize its limitations as per the elimination of NCDs. Exercise does not correct core microimmunosome issues creating the misregulated inflammation required for NCDs to exist/persist. Leaving the actual systems biology defects that cause and maintain NCDs fully in place in the population while expending global public health resources to pursue healthful, yet peripherally relevant, incomplete approaches to the NCD epidemic is, in the end, a net public disservice. It is important to note that once children are diagnosed with a NCD like asthma or obesity, double digit comorbidities will follow as the cohort ages [9]. This is problematic for ease of exercise beginning even in childhood. In 2016 the WHO published a report recognizing that NCDs have accompanying disabilities including physical limitations [160]. It is better never to start down the path of lifelong ever increasing NCDs.

In fact, if exercise were the complete route to stopping the NCD epidemic, then the WHO should be promoting ready microbiome-based strategies to gain the most out of exercise. For example, Lee et al. [161] showed that a single probiotic bacterium, human origin *Lactobacillus plantarum* PL-02 (obtained from the intestines of 2008 Olympic women’s 48 kg weightlifting gold medalist) 4-week supplementation could significantly increase muscle mass, muscle strength, endurance performance, and hepatic and muscular glycogen storage, while significantly decreasing lactate, blood urea nitrogen, ammonia, and creatine kinase. The take home message is that the microbiome should be included rather than excluded in virtually any initiative intended to benefit public health. Single-species humans do not now and probably never did exist. Public Health Institutions need to program and prioritize for the 21st century reality of humans as holobionts.

Beside the fact that the WHO’s exercise program does not go after the core causes and system biology defects propagating NCDs, it can be argued that the exercise program is also incomplete because it is too late in light of known developmental and transgenerational epigenetic programming of NCDs. As will be discussed in the following section, the reality is that early life is the window during which most NCDs become programmed within the life course [162,163].

### 16.1. Early-Life Programming of NCDs vs. Exercise

While exercise is a healthful pursuit as previously described, there is problematic reality mentioned earlier for Public Health entities like the WHO that are touting exercise as the conqueror of the NCD epidemic. The problem is the 1990s-originated science originally known as the Barker Hypothesis and later termed DOHaD. The Barker Hypothesis, originally described more than 30 years ago by British physician/researcher D.J. Barker, states that fetal and infant conditions can program for later life adult cardiovascular disease [164]. As more researchers examined this hypothesis, the windows of vulnerability for disease programming were expanded to include portions of childhood [56]. The number of diseases that can be programmed in early life expanded exponentially to include virtually all NCDs. 

A decade ago, Hanson and Glickman [165] called for a shift in public health policy to reflect the reality of early-life programming of the world’s number one killer, NCDs. However, there is little evidence to indicate that this shift in focus has actually happened. Certainly, the WHO’s four pillars to defeat the NCD epidemic (including exercise) does not seem to be oriented toward fetal and infant development.

### 16.2. Priority of the Microbiome and the First 1000 Days Concept

With the discovery of the importance of the microbiome for programmed development of the baby’s physiological systems (including the immune system) an even greater concern has arisen to protect and nurture early life of the human superorganism as the way to stop the NCD epidemic. The Barker Hypothesis, DOHaD, and the microbiome’s impact in early life was discussed previously by Dietert [9] in the preceding Microbiome First Medicine paper in this journal. The point was stressed that several key microbiome seeding- and feeding-related events must occur to rebalance the fetal immune system and prevent immune inflammation-inflicted NCDs as ageing occurs. Here, it is important to note the Public Health’s combined lack of priority for microbiome-aided infant development and correction of “regulatory gaps” in safety for the fetus and infant has done more to fuel the ongoing NCD epidemic than to bring it to an end.

With the microbiome added into DOHaD, the concept of a new overarching critical window of vulnerability has emerged: the First 1000 days of a baby’s life (fetal and neonatal). During the perinatal period of infant development starting with issues of pregnancy, mode of delivery, antibiotics, colostrum, and breastfeeding, there is a critical need to manage both mom’s and baby’s microbes. Simply put, the evidence suggests that public health attention to the whole superorganism is needed and the most gain in health protection and life-long health benefits per medical and the public health efforts is during a period of infant microbe-physiological programming that spans approximately the first 1000 days of a baby’s life [166,167,168]. 

Several pediatric-related groups and organizations have described the plasticity of the infant’s first 1000 days and called upon public health to make this the highest priority to prevent NCDs [169,170,171,172]. However, it is critical that the focus is not simply on the period of infant development. Focus should also be directed toward the microbiome, its impact on systems biology development, such as with the microimmunosome, the gut–immune–brain–axis, and the gut–bile salt metabolism interactions [9], as well as superorganism safety from toxic drugs, foods, food additives, and environmental chemicals. The First 1000 days focus is useful but only if it is human superorganism wide. Public health institutions like the WHO, FDA, NIH, EPA, USDA, CDC, and their equivalents in other countries need to refocus their NCD epidemic-fighting priorities to where our current science indicates it would be most effective: on the microbiome, particularly in early life. That is a path where public health can reverse its lengthy legacy of failures (Table 1).

## 17. Conclusions

By many accounts, public health organizations and institutions have taken a glorious beginning and turned it into a lengthy series of failures over the past half century. Organizations like the WHO have recognized that NCDs are the world’s number one killer and that a NCD epidemic has been raging for decades. Yet, despite public health initiatives, the numbers indicate that the epidemic is still raging unabated. This pattern of failure is understandable since public health priorities have been slow to embrace the overriding importance of the microbiome and the reality that: (1) many NCDs are programmed in early life, and (2) NCDs begin to emerge during childhood (e.g., childhood asthma, obesity) [9]. The four pillars for fighting NCDs chastise the public for poor nutrition, addictive behaviors, and inactivity. They stress that behavioral modification is the solution to our NCD epidemic [13]. This current review illustrates that this initiative is doomed to failure because it fails to include the need to manage the microbiome to readily change diet and withdrawal from food, drug, and chemical addiction. Chastising the public without providing critical microbiome-based education and tools required for individuals to pursue a healthy life is not a path to success regarding NCDs. At the same time an effort is needed to remove microbiome-damaging foods, drugs, food additives, and chemicals from our cities, store shelves, and households. Our past sins of not identifying and/or removing hazards for the human superorganism need to be corrected. The goal is not to damage the microbiome during pregnancy and the first 1000 days of infant development. Instead, it is to support mother and child beginning first and foremost with the majority of their cells and genes, the human microbiome.

## Figures and Tables

**Table 1:** Thirteen Examples Reflecting Outcome-Based Failures of Public Health and/or Major Public Health Initiatives that Produced Underwhelming Results.

Public Health Initiatives, Challenges, and Responses	Reference(s)
The World Health Organization recently tabulated that the vast majority of global deaths (71%) are caused by NCDs. However, they offer no plans seemingly capable of eliminating NCDs as the major cause of death.	[13]
The Global Burden of Disease Study illustrates the ongoing NCD epidemic but fails to even mention the microbiome among 87 risk factors and 369 diseases considered across hundreds of countries. It concluded that people are living more years in poor health despite medical advancements.	[19]
The extent of the failure of public health initiatives to address the decades-long NCD epidemic was revealed via a recent NHANES study survey. The study found that 91.8% of senior adults in the United States carry two or more NCDs.	[9,10]
The public health failure regarding the epidemic of multimorbid NCDs associated with aging was compounded when public health institutions failed to adequately protect the NCD-riddled, pro-inflammatory, and hyper-vulnerable geriatric population against the SARS-CoV-2-induced lethal cytokine storm.	[20,21,22]
Public health mandates during the SARS-CoV-2 pandemic that further eroded the human microbiome instead of protecting the microbiome and the microimmunosome.	[23,24]
The National Children’s Study was a grand 2000 Congressionally mandated, NIH-led inter-federal agency plan to prioritize early life health risk identification and prevention as the keys to better health both for children and across the lifespan. It was closed in 2014 with little to show for the very expensive initiative.	[25,26,27,28]
The Swine Flu incident beginning at Ft. Dix, NJ in 1976 and the rushed national vaccination program for a pandemic that never showed up proved to cause more health damage than good.	[29,30]
Public Health protection programs have repeatedly experienced “regulatory gaps” that permitted millions of people across multiple generations to be exposed to NCD-promoting toxicants before the hazard was eventually recognized. These safety testing gaps are not tied to a lack of microbiome safety evaluation. Examples of such global exposures to “safe” chemicals include: asbestos, trichloroethelene, dioxin, polychlorinated biphenyls, plasticizers including bisphenol A, atrazine, triclosan, perfluorinated compounds, microplastics, certain nanoparticles, and other endocrine disruptors and obesogens.	[31,32,33,34,35,36,37,38,39,40]
A plethora of food, food additives, drugs, and environmental chemicals previously approved by the FDA, the USDA, and the EPA have been shown to significantly damage the microimmunosome posing a significant risk to human health. Screening for microimmunsome safety would have been useful as would regulatory action based on identified toxicity for the microbiome.	[41,42,43,44,45,46,47]
Medical Journal calls to reverse the Public Health failure of the NCD epidemic has produced little effect.	[3]
The Human Genome Project was touted as the keystone through which most human diseases would be cured. Instead, it resulted in an underwhelming number of chromosomal genes identified and few diseases cured to date.	[48]
Risk–benefit decisions in Flint, MI resulted in years of exposure of children and adults to the neurotoxic, microimmunosome-damaging, heavy metal Pb (lead).	[4,49,50]
Disowning fundamental immunology and the role of natural immunity during the SARS-CoV-2 pandemic and the importance of T cell responses to viruses in heterologous adaptive immunity	[51,52,53]

**Table 2:** Microbiota and the Regulation of Taste.

Sense	Test Species/Group	Microbe(s) Involved/Subjects Discussed	Effects	Reference(s)
Taste	Human (with some mouse research brought in)	*Staphylococci, Streptococci Actinomyces, Lactobacillus Prevotella, Porphyromonas Actinobacteria and Bacteroidetes Actinomyces, Oribacterium, Solobacterium, Catonella, Campylobacter Clostridia Proteobacteria, Prevotella Streptococci mutans*	In this review article, these bacteria have been associated with changes in specific aspects of taste.	[96]
Taste	Human (emphasis on dental patients)	General review of broad scope on taste and including smell. The impact of biofilms is considered.	This review emphasizes the life course ramification of flavor biases and the potential risk to the aging population.	[97]
Taste thresholds	Human (preschool children)	Oral microbiota affecting sweet taste thresholds in children	This is an important study showing that preschool with a lower threshold for perceiving sugar consumed less sugar, had fewer dental caries, and a general lack of oral *Streptococcus mutans.* The reverse was true for children with high thresholds for perceiving sugar.	[98]
Taste	Human (adults and youth)	This was a crowdsourced population study of adults and youth. *Treponema* was found in the oral microbiome of adults with dental problems and of obese youth.	The observation of *Treponema* in youth suggests it might be a biomarker for later oral health problems and connected in some way to the childhood obesity. This study did not find a microbial sweetness taste difference among the crowdsourced sampling.	[99]
Taste	Human	Oral-tongue review of how the mouth microbiome affects the gut microbiome, barrier integrity, inflammation, indirectly the gut–brain axis, the liver and all through taste regulation	The tongue microbiome and its dysbiosis can be a large contributor to metabolic disorders that facilitate obesity, diabetes, and cardiovascular disease.	[100]
Taste	Human (dental patients, teenagers and young adults)	Oral microbiota were characterized among dental patients with differing sugar intake and caries vs. fewer caries	Specific oral microbiota were associated with sugar intake. However, there were several distinct ecological combinations of microbiota that were associated with high sugar intake.	[101]
Taste	Human (men)	Examined obese men for oral microbiota signatures within the circumvallate papillae (CVP) that relate to fatty taste perception.	Decreased fatty taste perception was associated with elevated *Bacteroides genus* and *Clostridium_XIV* and decreased *Lactobacillus* compared against the high fatty taste perception group.	[102]
Taste	Human (diabetic patients)	Examined type 2 diabetic patients for oral microbiota signatures within the circumvallate papillae (CVP) that relate to fatty taste perception.	Impaired fatty acid perception is not driven by insulin resistance but rather is affected by microbiota dysbiosis. Additionally, some drugs (e.g., metformin, statins) may affect lipid sensitivity perception	[103]
Taste thresholds and intensity	Human	Analysis of orosensory perception of lipids and sweets in adult females following different types of gastric surgery	Low numbers of patients and high individual variability produced few statistically significant differences beyond a microbiome signature.	[104]
Taste sensitivity	Human	This is a review article examining fat taste sensitivity and microbiota.	The article focuses on insensitivity to long-chain dietary fatty acids, the microbiota that are associated with reduced dietary fat detection, and this physiological–microbiological change as a path to obesity.	[105]
Taste distinctions	Human	Taste perception, oral microbiota, and childhood obesity were compared in the cross-sectional study	In this cross-sectional study, obese children vs. controls had difficulty identifying taste quality. A lower number of Fungiform Papillae, a lower oral microbiome alpha diversity, and some subtle differences in microbiota representation were reported.	[106]
Taste perceptions and food preferences	Human	Oral microbiota, perceptions, and dietary preferences	In a study of 59 volunteers, the results indicated a correlation between tongue dorsum microbiota, gustatory function, and specific food intake. The *Clostridia* class was associated with high energy, protein, and fat intake while *Prevotalla* genus bacteria were associated with high fiber intake.	[107]
Taste/Eating Behaviors	Rat	Maternal microbiota program the offspring’s eating behavior.	Maternal microbiota transfer from obese prone or obese resistance dams into F344 strain neonates helped to establish that neonatal microbiota can program juveniles and adult eating behaviors. This programming did not require the transferred microbiota to persist into adulthood. It is a microbiome-based example of DOHaD.	[108]
Taste Perception	Mouse	Prebiotic modulates sweet taste perception in obese mice	An inulin-type fructan prebiotic was administered to diet-induced obese mice for 12 weeks. The supplementation produced an elevation of cecal *Bifidobacteria* and *Akkermansia* and improved the orosensory perception of sweet compounds.	[109]
Taste/Food choice behavior	Drosophila research model study	Ingestion of foreign microbiota produced a strong shift in dietary preferences.	A strong food aversion was evolved into a strong food preference by repeated ingestion of microbiota derived from a different *Drosophila* species.	[110]
Taste/Food choice behavior	Drosophila research model study	Strong dietary preferences controlled by the metabolism of commensal bacteria.	Commensal bacteria were shown to direct food preferences via metabolic activity and could overcome some direct effects of the food itself.	[111]
Taste and Smell	Review	Oral microbiota metabolism affects flavor perception thresholds via multiple routes. Taste and smell perceptions are both affected.	Comprehensive coverage of the multiple pathways through which both taste and smell are affected by microbiota.	[112]

**Table 3:** The Role of Microbiota in Smell.

Sense	Test Species/Group	Microbe(s) Involved/Subjects Discussed	Effects	Reference(s)
Smell	Mouse (three groups)	Characterization of microbiota among three distinct groups of mice: C3H/HeN, Swiss, and BALB/cByJ. Germ free mice (C3H/HeN) were installed with microbiota from each of the three groups permitting olfactory, electro-olfactogram recordings (EOG) of the epithelium and microbiota comparisons on the same mouse genetic background.	Among 11 odorants examined, several differentially activated the olfactory epithelium linked to the microbiota profile. The findings suggest the importance of the microbiota in the olfactory epithelium physiology (e.g., EOG).	[115]
Smell	Mouse and Human (Review)	Chemosensory links between microbiota, olfaction and emotion are described and the “odorome” concept is introduced. An example of bacterial products discussed is β-phenylethylamine.	This is an important review article covering the capacity of bacterial products to affect olfactory receptors and, in turn, to elicit specific emotions.	[116]
Smell	Human	Bacterial signatures from among *Actinobacteria, Bacteroidia, Bacilli, Clostridia and* *Proteobacteria* were associated with hyposmic (i.e., odor detection) threshold, low discrimination and low identification performance. *Corynebacterium* and *Faecalibacterium* were often biomarkers for reduced odor discrimination and threshold. *Comamonadaceae* and *Enterobacteriaceae* were linked with reduce thresholds and identification. *Porphyromonas* and unclassified *Lachnospiraceae* were associated with poor performance across all three olfactory performance categories.	Odor thresholds, identification and discrimination were all evaluated and found to be linked by microbiota composition. Specific groups of microbiota affected combinations of the three categories.	[117]
Smell	Mice and Zebrafish	Mechanistic study in two animal models	Found evidence for nasal microbiota regulation of olfactory transcriptional factors	[118]

**Table 4:** The Role of Microbiota in Satiety.

Sense	Test Species/Group	Microbe(s) Involved/Subjects Discussed	Effects	Reference(s)
Satiety	Review of multi-species studies (primarily rodent with some human)	This recent review article details the variety of mechanisms through which gut microbiota control satiety	Microbiota were demonstrated to control both central and peripheral food intake mechanisms.	[121]
Satiety	Review of human and mouse studies	Review article covering probiotic and prebiotic studies on eating and satiety. It also discusses the categories of microbial peptides, hormones, and products as well as metabolites that affect hunger, eating, and satiety. Most of the probiotic studies cited used *Lactobacillus* and/or *Bifidobacterium* species.	This review describes the control of multiple regulatory factors affecting satiety that are embedded within the gut microbiome. It also summarizes numerous clinical and research studies on microbiota and appetite control.	[122]
Satiety	Review	Review article covering microbiota–gut–brain axis in satiety regulation	The article is focused on how we move toward microbiota–gut–brain axis on a chip in vitro assessment.	[123]
Satiety	Review	Review article detailing the regulation of gut peptides and particularly ghrelin via gut microbiota	This review article provides satiety-related evidence that gut microbiota regulates ghrelin levels via short chain fatty acids, specific amino acids, formyl peptides, LPS, and H2S, and affects ghrelin receptor signaling.	[124]
Satiety	Obese adults	*Lactobacillus rhamnosus* CGMCC1.3724 (LPR)	This was a 24-week duration (two 12-week phases) double-blind, randomized, placebo-controlled trial examining control of appetite, weight loss, and mood. Positive significant effects on satiety were seen in both men and woman with the latter experiencing the greater benefit.	[125]
Satiety	Obese women	A multi-species probiotic mix or placebo was used in combination with a caloric restricted diet. The probiotic mix contained: *Lactobacillus acidophilus, Bifidobacterium bifidum*, *Bifidobacterium lactis*, *Bifidobacterium longum*, *Lactobacillus rhamnosus,* *Lactobacillus reuteri*, magnesium stearate, and maltodextrin	This was a 12-week duration, randomized, double-blind, placebo-controlled clinical trial of obese women. Positive effects were seen in the probiotic supplemented group for both eating behavior as well as anthropometric indices.	[126]
Satiety	Mouse	*Lactobacillus rhamnosus* GG	Aged Balb/c mice were fed a regular diet or a high fat diet and two different doses of probiotic supplementation were examined for effects on obesity-related biomarkers including leptin resistance. High dose probiotic reversed the leptin-resistance associated with diet-induced obesity.	[127]
Satiety	Mouse	A prebiotic soybean insoluble dietary fiber was administered during a 24-week intervention in high fat diet mice. The specialized fiber induced increases in *Lactobacillus* and *Lachnospirace*_Nk4A136_group with decreases in *Lachnospiraceae* and *Bacteroidesacidifaciens*	The outcomes of the 24-week prebiotics intervention and microbiome shift were changes in short chain fatty acid production and an elevation in satiety hormones.	[128]

## References

[1] Duffy J. (1992). The Sanitarians: A History of American Public Health.

[2] Cranor C.F. (2017). Tragic Failures: How and Why We are Harmed by Toxic Chemicals.

[3] Editorial (2004). The catastrophic failures of public health. Lancet.

[4] Bellinger D.C. (2016). Lead Contamination in Flint—An Abject Failure to Protect Public Health. N. Engl. J. Med..

[5] Maffini M.V., Neltner T.G., Vogel S. (2017). We are what we eat: Regulatory gaps in the United States that put our health at risk. PLoS Biol..

[6] Myers J.P., Antoniou M.N., Blumberg B., Carroll L., Colborn T., Everett L.G., Hansen M., Landrigan P.J., Lanphear B.P., Mesnage R. (2016). Concerns over use of glyphosate-based herbicides and risks associated with exposures: A consensus statement. Environ. Health.

[7] Lobstein T., Brownell K.D. (2021). Endocrine-disrupting chemicals and obesity risk: A review of recommendations for obesity prevention policies. Obes. Rev..

[8] Wilkinson J.E., Franzosa E.A., Everett C., Li C., Hu F.B., Wirth D.F., Song M., Chan A.T., HCMPH Researchers and Trainees, HCMPH Investigators (2021). A framework for microbiome science in public health. Nat. Med..

[9] Dietert R.R. (2021). Microbiome First Medicine in Health and Safety. Biomedicines.

[10] King D.E., Xiang J., Pilkerton C.S. (2018). Multimorbidity Trends in United States Adults, 1988–2014. J. Am. Board Fam. Med..

[11] Bloom D.E., Cafiero E.T., Jané-Llopis E., Abrahams-Gessel S., Bloom L.R., Fathima S., Feigl B., Gaziano T., Mowafi M., Pandya A. (2011). The Global Economic Burden of Noncommunicable Diseases.

[12] World Health Organization (2021). Noncommunicable Diseases Fact Sheet.

[13] World Health Organization Preventing Noncommunicable Diseases. https://www.who.int/activities/preventing-noncommunicable-diseases.

[14] Newsholme A. (1903). The possible association of the consumption of alcohol with excessive mortality from cancer. Br. Med. J..

[15] The Tobacco Heart. The Hospital 1901, 30, 6. https://www.ncbi.nlm.nih.gov/pmc/articles/PMC5186887/?page=1.

[16] Foxcroft L. (2012). Calories and Corsets: A History of Dieting over Two Thousand Years.

[17] Tipton C.M. (2014). The history of “Exercise Is Medicine” in ancient civilizations. Adv. Physiol. Educ..

[18] Franklin B., Matthews B. (1914). Dialog Between Franklin and the Gout. The Oxford Book of American Essays.

[19] GBD 2019 Risk Factors Collaborators 2020 (2020). Global burden of 87 risk factors in 204 countries and territories, 1990–2019: A systematic analysis for the Global Burden of Disease Study 2019. Lancet.

[20] Dean E., Skinner M., Yu H.P., Jones A.Y., Gosselink R., Söderlund A. (2021). Why COVID-19 strengthens the case to scale up assault on non-communicable diseases: Role of health professionals including physical therapists in mitigating pandemic waves. AIMS Public Health.

[21] Dietert R.R. (2021). Lessons for human holobiont medicine in the era of SARS-Cov-2. Am. J. Biomed. Sci. Res..

[22] Chan E.Y.Y., Kim J.H., Lo E.S.K., Huang Z., Hung H., Hung K.K.C., Wong E., Lee E.K.P., Wong M.C.S., Wong S.Y.S. (2020). What Happened to People with Non-Communicable Diseases during COVID-19: Implications of H-EDRM Policies. Int. J. Environ. Res. Public Health.

[23] Finlay B.B., Amato K.R., Azad M., Blaser M.J., Bosch T.C.G., Chu H., Dominguez-Bello M.G., Ehrlich S.D., Elinav E., Geva-Zatorsky N. (2021). The hygiene hypothesis, the COVID pandemic, and consequences for the human microbiome. Proc. Natl. Acad. Sci. USA.

[24] Dietert R.R. (2021). The microbiological basis of human superorganism freedom. Am. J. Biomed. Sci. Res..

[25] Office of the Director—Eunice Kennedy Shriver National Institute of Child Health and Human Development National Children’s Study Archive (version 5/12/20). https://www.nichd.nih.gov/research/supported/NCS.

[26] Hudak M.L., Park C.H., Annett R.D., Hale D.E., McGovern P.M., McLaughlin T.J., Dole N., Kaar J.L., Balsam M.J. (2016). The National Children’s Study: An Introduction and Historical Overview. Pediatrics.

[27] Kaiser J. (2014). NIH Cancels Massive U.S. Children’s Study. Science. https://www.science.org/news/2014/12/nih-cancels-massive-us-children-s-study.

[28] Schmidt C. (2016). The Death of the National Children’s Study: What Went Wrong? Undark. https://undark.org/2016/05/25/the-death-of-a-study-national-childrens-study/.

[29] Greenstreet R. (1983). Adjustment of Rates of Guillain-Barré Syndrome among Recipients of Swine Flu Vaccine, 1976–1977. J. R. Soc. Med..

[30] Sencer D.J., Millar J.D. (2006). Reflections on the 1976 swine flu vaccination program. Emerg. Infect. Dis..

[31] Arachi D., Furuya S., David A., Mangwiro A., Chimed-Ochir O., Lee K., Tighe P., Takala J., Driscoll T., Takahashi K. (2021). Development of the “National Asbestos Profile” to Eliminate Asbestos-Related Diseases in 195 Countries. Int. J. Environ. Res. Public Health.

[32] Banerjee N., Wang H., Wang G., Boor P.J., Khan M.F. (2021). Redox-sensitive Nrf2 and MAPK signaling pathways contribute to trichloroethene-mediated autoimmune disease progression. Toxicology.

[33] Ben Maamar M., Nilssonm E., Thorson J.L.M., Beck D., Skinner M.K. (2021). Transgenerational disease specific epigenetic sperm biomarkers after ancestral exposure to dioxin. Environ. Res..

[34] McCann M.S., Fernandez H.R., Flowers S.A., Maguire-Zeiss K.A. (2021). Polychlorinated biphenyls induce oxidative stress and metabolic responses in astrocytes. Neurotoxicology.

[35] Pirozzi C., Lama A., Annunziata C., Cavaliere G., Ruiz-Fernandez C., Monnolo A., Comella F., Gualillo O., Stornaiuolo M., Mollica M.P. (2020). Oral Bisphenol A Worsens Liver Immune-Metabolic and Mitochondrial Dysfunction Induced by High-Fat Diet in Adult Mice: Cross-Talk between Oxidative Stress and Inflammasome Pathway. Antioxidants.

[36] Pandey N., Maske P., Mote C., Dighe V. (2021). Exposure to Atrazine through gestation and lactation period led to impaired sexual maturation and subfertility in F1 male rats with congenital deformities in F2 progeny. Food Chem. Toxicol..

[37] Wang D., Liu J., Jiang H. (2021). Triclosan regulates the Nrf2/HO-1 pathway through the PI3K/Akt/JNK signaling cascade to induce oxidative damage in neurons. Environ. Toxicol..

[38] Qiu L., Wang H., Dong T., Huang J., Li T., Ren H., Wang X., Qu J., Wang S. (2021). Perfluorooctane sulfonate (PFOS) disrupts testosterone biosynthesis via CREB/CRTC2/StAR signaling pathway in Leydig cells. Toxicology.

[39] Kannan K., Vimalkuma K. (2021). A Review of Human Exposure to Microplastics and Insights Into Microplastics as Obesogens. Front. Endocrinol..

[40] Chen L., Nie P., Yao L., Tang Y., Hong W., Liu W., Fu F., Xu H. (2021). TiO2 NPs induce the reproductive toxicity in mice with gestational diabetes mellitus through the effects on the endoplasmic reticulum stress signaling pathway. Ecotoxicol. Environ. Saf..

[41] Hu J., Lesseur C., Miao Y., Manservisi F., Panzacchi S., Mandrioli D., Belpoggi F., Chen J., Petrick L. (2021). Low-dose exposure of glyphosate-based herbicides disrupt the urine metabolome and its interaction with gut microbiota. Sci. Rep..

[42] Le Bastard Q., Berthelot L., Soulillou J.P., Montassier E. (2021). Impact of non-antibiotic drugs on the human intestinal microbiome. Expert Rev. Mol. Diagn..

[43] Wang X., Tang Q., Hou H., Zhang W., Li M., Chen D., Gu Y., Wang B., Hou J., Liu Y. (2021). Gut Microbiota in NSAID Enteropathy: New Insights From Inside. Front. Cell Infect. Microbiol..

[44] Inghammar M., Svanström H., Voldstedlund M., Melbye M., Hviid A., Mølbak K., Pasterna B. (2021). Proton-Pump Inhibitor Use and the Risk of Community-Associated Clostridium difficile Infection. Clin. Infect. Dis..

[45] Bancil A.S., Sandall A.M., Rossi M., Chassaing B., Lindsay J.O., Whelan K. (2021). Food Additive Emulsifiers and Their Impact on Gut Microbiome, Permeability, and Inflammation: Mechanistic Insights in Inflammatory Bowel Disease. J. Crohns Colitis.

[46] Naimi S., Viennois E., Gewirtz A.T., Chassaing B. (2021). Direct impact of commonly used dietary emulsifiers on human gut microbiota. Microbiome.

[47] Jin G., Tang Q., Ma J., Liu X., Zhou B., Sun Y., Pang X., Guo Z., Xie R., Liu T. (2021). Maternal Emulsifier P80 Intake Induces Gut Dysbiosis in Offspring and Increases Their Susceptibility to Colitis in Adulthood. mSystems.

[48] Latham J. (2011). The Failure of the Genome. https://www.theguardian.com/commentisfree/2011/apr/17/human-genome-genetics-twin-studies.

[49] DeWitt R.D. (2017). Pediatric lead exposure and the water crisis in Flint, Michigan. J. Am. Acad. PAs.

[50] Hanna-Attisha M., Lanphear B., Landrigan P. (2018). Lead Poisoning in the 21st Century: The Silent Epidemic Continues. Am. J. Public Health.

[51] Block J. (2021). Vaccinating people who have had covid-19: Why doesn’t natural immunity count in the US?. BMJ.

[52] Bejarano D.A., Schlitzer A. (2021). Breathing more breadth into COVID-19 T cell responses. Med.

[53] CDC Statement (2021). Natural Immunity and Vaccinations. CDC Media Relations. https://www.cdc.gov/media/releases/2021/s0806-vaccination-protection.html.

[54] Zabetakis I., Lordan R., Norton C., Tsoupras A. (2020). COVID-19: The Inflammation Link and the Role of Nutrition in Potential Mitigation. Nutrients.

[55] Nikoloski Z., Alqunaibet A.M., Alfawaz R.A., Almudarra S.S., Herbst C.H., El-Saharty S., Alsukait R., Algwizani A. (2021). Covid-19 and non-communicable diseases: Evidence from a systematic literature review. BMC Public Health.

[56] Dietert R.R., Etzel R.A., Chen D., Halonen M., Holladay S.D., Jarabek A.M., Landreth K., Peden D.B., Pinkerton K., Smialowicz R.J. (2000). Workshop to identify critical windows of exposure for children’s health: Immune and respiratory systems work group summary. Environ. Health Perspect..

[57] Barouki R., Gluckman P.D., Grandjean P., Hanson M., Heindel J.J. (2012). Developmental origins of non-communicable disease: Implications for research and public health. Environ. Health.

[58] Anway M.D., Skinner M.K. (2006). Epigenetic transgenerational actions of endocrine disruptors. Endocrinology.

[59] George F., Mahieux S., Daniel C., Titécat M., Beauval N., Houcke I., Neut C., Allorge D., Borges F., Jan G. (2021). Assessment of Pb(II), Cd(II), and Al(III) Removal Capacity of Bacteria from Food and Gut Ecological Niches: Insights into Biodiversity to Limit Intestinal Biodisponibility of Toxic Metals. Microorganisms.

[60] Duan H., Yu L., Tian F., Zhai Q., Fan L., Chen W. (2020). Gut microbiota: A target for heavy metal toxicity and a probiotic protective strategy. Sci. Total Environ..

[61] Su H., Liu J., Wu G., Long Z., Fan J., Xu Z., Liu J., Yu Z., Cao M., Liao N. (2020). Homeostasis of gut microbiota protects against polychlorinated biphenyl 126-induced metabolic dysfunction in liver of mice. Sci. Total Environ..

[62] Yu L., Zhang L., Duan H., Zhao R., Xiao Y., Guo M., Zhao J., Zhang H., Chen W., Tian F. (2021). The Protection of Lactiplantibacillus plantarum CCFM8661 Against Benzopyrene-Induced Toxicity via Regulation of the Gut Microbiota. Front. Immunol..

[63] Dietert R.R., Dietert J.M. (2021). Twentieth Century Dogmas Prevent Sustainable Healthcare. Am. J. Biomed. Sci. Res..

[64] Cani P.D., Everard A. (2015). Keeping gut lining at bay: Impact of emulsifiers. Trends Endocrinol. Metab..

[65] Jiang Z., Zhao M., Zhang H., Li Y., Liu M., Feng F. (2018). Antimicrobial Emulsifier-Glycerol Monolaurate Induces Metabolic Syndrome, Gut Microbiota Dysbiosis, and Systemic Low-Grade Inflammation in Low-Fat Diet Fed Mice. Mol. Nutr. Food Res..

[66] Godinez Puig L., Lusk K., Glick D., Einstein K.L., Palmer M., Fox S., Wang M.L. (2021). Perceptions of Public Health Priorities and Accountability Among US Mayors. Public Health Rep..

[67] WHO Obesity and Overweight (2021). Fact Sheet. https://www.who.int/news-room/fact-sheets/detail/obesity-and-overweight.

[68] Vogel S.A. (2009). The politics of plastics: The making and unmaking of bisphenol a “safety”. Am. J. Public Health.

[69] Andújar N., Gálvez-Ontiveros Y., Zafra-Gómez A., Rodrigo L., Álvarez-Cubero M.J., Aguilera M., Monteagudo C., Rivas A.A. (2019). Bisphenol A Analogues in Food and Their Hormonal and Obesogenic Effects: A Review. Nutrients.

[70] Desai M., Ferrini M.G., Jellyman J.K., Han G., Ross M.G. (2018). In vivo and in vitro bisphenol A exposure effects on adiposity. J. Dev. Orig. Health Dis..

[71] Pérez-Bermejo M., Mas-Pérez I., Murillo-Llorente M.T. (2021). The Role of the Bisphenol A in Diabetes and Obesity. Biomedicines.

[72] Linares R., Fernández M.F., Gutiérrez A., García-Villalba R., Suárez B., Zapater P., Martínez-Blázquez J.A., Caparrós E., Tomás-Barberán F.A., Francés R. (2021). Endocrine disruption in Crohn’s disease: Bisphenol A enhances systemic inflammatory response in patients with gut barrier translocation of dysbiotic microbiota products. FASEB J..

[73] Roberts L. (1989). Genome Mapping Goal Now in Reach: James Watson has promised to complete a map of the human genome within 5 years; now it looks like it might be doable. Science.

[74] Anderson G.C. (1990). Human genome project. The honeymoon is over. Nature.

[75] Saey T.H. (2010). More than a Chicken, Fewer than a Grape. https://www.sciencenews.org/article/more-chicken-fewer-grape.

[76] Craft-Blacksheare M.G. (2017). Lessons Learned From the Crisis in Flint, Michigan Regarding the Effects of Contaminated Water on Maternal and Child Health. J. Obstet. Gynecol. Neonatal Nurs..

[77] Akdis C.A. (2021). Does the epithelial barrier hypothesis explain the increase in allergy, autoimmunity and other chronic conditions?. Nat. Rev. Immunol..

[78] Swaney M.H., Kalan L.R. (2021). Living in Your Skin: Microbes, Molecules, and Mechanisms. Infect. Immun..

[79] Nakatsuji T., Chen T.H., Narala S., Chun K.A., Two A.M., Yun T., Shafiq F., Kotol P.F., Bouslimani A., Melnik A.V. (2017). Antimicrobials from human skin commensal bacteria protect against *Staphylococcus aureus* and are deficient in atopic dermatitis. Sci. Transl. Med..

[80] Tan-Lim C.S.C., Esteban-Ipac N.A.R., Recto M.S.T., Castor M.A.R., Casis-Hao R.J., Nano A.L.M. (2021). Comparative effectiveness of probiotic strains on the prevention of pediatric atopic dermatitis: A systematic review and network meta-analysis. Pediatr. Allergy Immunol..

[81] Cukrowska B., Ceregra A., Maciorkowska E., Surowska B., Zegadło-Mylik M.A., Konopka E., Trojanowska I., Zakrzewska M., Bierła J.B., Zakrzewski M. (2021). The Effectiveness of Probiotic *Lactobacillus rhamnosus* and *Lactobacillus casei* Strains in Children with Atopic Dermatitis and Cow’s Milk Protein Allergy: A Multicenter, Randomized, Double Blind, Placebo Controlled Study. Nutrients.

[82] Callewaert C., Knödlseder N., Karoglan A., Güel M., Paetzold B. (2021). Skin microbiome transplantation and manipulation: Current state of the art. Comput. Struct. Biotechnol. J..

[83] Abuqwider J.N., Mauriello G., Altamimi M. (2021). *Akkermansia muciniphila*, a New Generation of Beneficial Microbiota in Modulating Obesity: A Systematic Review. Microorganisms.

[84] Ouyang J., Lin J., Isnard S., Fombuena B., Peng X., Marette A., Routy B., Messaoudene M., Chen Y., Routy J.P. (2020). The Bacterium *Akkermansia muciniphila*: A Sentinel for Gut Permeability and Its Relevance to HIV-Related Inflammation. Front. Immunol..

[85] Ottman N., Reunanen J., Meijerink M., Pietilä T.E., Kainulainen V., Klievink J., Huuskonen L., Aalvink S., Skurnik M., Boeren S. (2017). Pili-like proteins of *Akkermansia muciniphila* modulate host immune responses and gut barrier function. PLoS ONE.

[86] Anhê F.F., Pilon G., Roy D., Desjardins Y., Levy E., Marette A. (2016). Triggering *Akkermansia* with dietary polyphenols: A new weapon to combat the metabolic syndrome?. Gut Microbes.

[87] Huang D., Gao J., Li C., Nong C., Huang W., Zheng X., Li S., Peng Y. (2021). A potential probiotic bacterium for antipsychotic-induced metabolic syndrome: Mechanisms underpinning how *Akkermansia muciniphila* subtype improves olanzapine-induced glucose homeostasis in mice. Psychopharmacology.

[88] Carlier F.M., de Fays C., Pilette C. (2021). Epithelial Barrier Dysfunction in Chronic Respiratory Diseases. Front. Physiol..

[89] Nino G., Rodriguez-Martinez C.E., Gutierrez M.J. (2021). Early Microbial-Immune Interactions and Innate Immune Training of the Respiratory System during Health and Disease. Children.

[90] Coleman M., Dietert R.R., North D.W., Stephenson M.M. (2021). Enhancing Human Superorganism Ecosystem Resilience by Holistically ‘Managing our Microbes’. J. Appl. Microbiol..

[91] Nordengrün M., Abdurrahman G., Treffon J., Wächter H., Kahl B.C., Bröker B.M. (2021). Allergic Reactions to Serine Protease-Like Proteins of *Staphylococcus aureus*. Front. Immunol..

[92] Lokken-Toyli K.L., de Steenhuijsen Piters W.A.A., Zangari T., Martel R., Kuipers K., Shopsin B., Loomis C., Bogaert D., Weiser J.N. (2021). Decreased production of epithelial-derived antimicrobial molecules at mucosal barriers during early life. Mucosal Immunol..

[93] Cole A.L., Sundar M., Lopez A., Forsman A., Yooseph S., Cole A.M. (2021). Identification of Nasal Gammaproteobacteria with Potent Activity against *Staphylococcus aureus*: Novel Insights into the “Noncarrier” State. mSphere.

[94] Accorsi E.K., Franzosa E.A., Hsu T., Joice Cordy R., Maayan-Metzger A., Jaber H., Reiss-Mandel A., Kline M., DuLong C., Lipsitch M. (2020). Determinants of *Staphylococcus aureus* carriage in the developing infant nasal microbiome. Genome Biol..

[95] Liu Q., Liu Q., Meng H., Lv H., Liu Y., Liu J., Wang H., He L., Qin J., Wang Y. (2020). *Staphylococcus* epidermidis Contributes to Healthy Maturation of the Nasal Microbiome by Stimulating Antimicrobial Peptide Production. Cell Host Microbe.

[96] Leung R., Covasa M. (2021). Do Gut Microbes Taste?. Nutrients.

[97] Ellender G., Moynihan P. (2021). Oral Health Impacts on Flavor and Significance in Dental Treatment. JDR Clin. Trans. Res..

[98] Jurczak A., Jamka-Kasprzyk M., Bębenek Z., Staszczyk M., Jagielski P., Kościelniak D., Gregorczyk-Maga I., Kołodziej I., Kępisty M., Kukurba-Setkowicz M. (2020). Differences in Sweet Taste Perception and Its Association with the *Streptococcus mutans* Cariogenic Profile in Preschool Children with Caries. Nutrients.

[99] Burcham Z.M., Garneau N.L., Comstock S.S., Tucker R.M., Knight R., Metcalf J.L., Genetics of Taste Lab Citizen Scientists (2020). Patterns of Oral Microbiota Diversity in Adults and Children: A Crowdsourced Population Study. Sci. Rep..

[100] Li Y., Cui J., Liu Y., Chen K., Huang L., Liu Y. (2021). Oral, Tongue-Coating Microbiota, and Metabolic Disorders: A Novel Area of Interactive Research. Front. Cardiovasc. Med..

[101] Esberg A., Haworth S., Hasslöf P., Lif Holgerson P., Johansson I. (2020). Oral Microbiota Profile Associates with Sugar Intake and Taste Preference Genes. Nutrients.

[102] Besnard P., Christensen J.E., Bernard A., Collet X., Verges B., Burcelin R. (2020). Fatty taste variability in obese subjects: The oral microbiota hypothesis. Oilseeds Fats Crop. Lipids.

[103] Besnard P., Christensen J.E., Bernard A., Simoneau-Robin I., Collet X., Verges B., Burcelin R. (2020). Identification of an oral microbiota signature associated with an impaired orosensory perception of lipids in insulin-resistant patients. Acta Diabetol..

[104] Bernard A., Le Beyec-Le Bihan J., Radoi L., Coupaye M., Sami O., Casanova N., Le May C., Collet X., Delaby P., Le Bourgot C. (2021). Orosensory Perception of Fat/Sweet Stimuli and Appetite-Regulating Peptides before and after Sleeve Gastrectomy or Gastric Bypass in Adult Women with Obesity. Nutrients.

[105] Khan A.S., Keast R., Khan N.A. (2020). Preference for dietary fat: From detection to disease. Prog. Lipid Res..

[106] Mameli C., Cattaneo C., Panelli S., Comandatore F., Sangiorgio A., Bedogni G., Bandi C., Zuccotti G., Pagliarini E. (2019). Taste perception and oral microbiota are associated with obesity in children and adolescents. PLoS ONE.

[107] Cattaneo C., Riso P., Laureati M., Gargari G., Pagliarini E. (2019). Exploring Associations between Interindividual Differences in Taste Perception, Oral Microbiota Composition, and Reported Food Intake. Nutrients.

[108] Pocheron A.L., Le Dréan G., Billard H., Moyon T., Pagniez A., Heberden C., Le Chatelier E., Darmaun D., Michel C., Parnet P. (2021). Maternal Microbiota Transfer Programs Offspring Eating Behavior. Front. Microbiol..

[109] Bernard A., Ancel D., Neyrinck A.M., Dastugue A., Bindels L.B., Delzenne N.M., Besnard P. (2019). A Preventive Prebiotic Supplementation Improves the Sweet Taste Perception in Diet-Induced Obese Mice. Nutrients.

[110] Heys C., Fisher A.M., Dewhurst A.D., Lewis Z., Lizé A. (2021). Exposure to foreign gut microbiota can facilitate rapid dietary shifts. Sci. Rep..

[111] Leitão-Gonçalves R., Carvalho-Santos Z., Francisco A.P., Fioreze G.T., Anjos M., Baltazar C., Elias A.P., Itskov P.M., Piper M.D.W., Ribeiro C. (2017). Commensal bacteria and essential amino acids control food choice behavior and reproduction. PLoS Biol..

[112] Schwartz M., Canon F., Feron G., Neiers F., Gamero A. (2021). Impact of Oral Microbiota on Flavor Perception: From Food Processing to In-Mouth Metabolization. Foods.

[113] Thompson M.D., DeBosch B.J. (2021). Maternal Fructose Diet-Induced Developmental Programming. Nutrients.

[114] Beisner J., Gonzalez-Granda A., Basrai M., Damms-Machado A., Bischoff S.C. (2020). Fructose-Induced Intestinal Microbiota Shift Following Two Types of Short-Term High-Fructose Dietary Phases. Nutrients.

[115] Naudon L., François A., Mariadassou M., Monnoye M., Philippe C., Bruneau A., Dussauze M., Rué O., Rabot S., Meunier N. (2020). First step of odorant detection in the olfactory epithelium and olfactory preferences differ according to the microbiota profile in mice. Behav. Brain Res..

[116] Bienenstock J., Kunze W.A., Forsythe P. (2018). Disruptive physiology: Olfaction and the microbiome-gut-brain axis. Biol. Rev. Camb. Philos Soc..

[117] Koskinen K., Reichert J.L., Hoier S., Schachenreiter J., Duller S., Moissl-Eichinger C., Schöpf V. (2018). The nasal microbiome mirrors and potentially shapes olfactory function. Sci. Rep..

[118] Casadei E., Tacchi L., Lickwar C.R., Espenschied S.T., Davison J.M., Muñoz P., Rawls J.F., Salinas I. (2019). Commensal Bacteria Regulate Gene Expression and Differentiation in Vertebrate Olfactory Systems Through Transcription Factor REST. Chem. Senses.

[119] Morquecho-Campos P., de Graaf K., Boesveldt S. (2020). Smelling our appetite? The influence of food odors on congruent appetite, food preferences and intake. Food Qual. Prefer..

[120] Uauy R., Díaz E. (2005). Consequences of food energy excess and positive energy balance. Public Health Nutr..

[121] Rautmann A.W., de La Serre C.B. (2021). Microbiota’s Role in Diet-Driven Alterations in Food Intake: Satiety, Energy Balance, and Reward. Nutrients.

[122] Han H., Yi B., Zhong R., Wang M., Zhang S., Ma J., Yin Y., Yin J., Chen L., Zhang H. (2021). From gut microbiota to host appetite: Gut microbiota-derived metabolites as key regulators. Microbiome.

[123] Pizarroso N.A., Fuciños P., Gonçalves C., Pastrana L., Amado I.R. (2021). A Review on the Role of Food-Derived Bioactive Molecules and the Microbiota-Gut-Brain Axis in Satiety Regulation. Nutrients.

[124] Leeuwendaal N.K., Cryan J.F., Schellekens H. (2021). Gut peptides and the microbiome: Focus on ghrelin. Curr. Opin. Endocrinol. Diabetes Obes..

[125] Sanchez M., Darimont C., Panahi S., Drapeau V., Marette A., Taylor V.H., Doré J., Tremblay A. (2017). Effects of a Diet-Based Weight-Reducing Program with Probiotic Supplementation on Satiety Efficiency, Eating Behaviour Traits, and Psychosocial Behaviours in Obese Individuals. Nutrients.

[126] Narmaki E., Borazjani M., Ataie-Jafari A., Hariri N., Doost A.H., Qorbani M., Saidpour A. (2020). The combined effects of probiotics and restricted calorie diet on the anthropometric indices, eating behavior, and hormone levels of obese women with food addiction: A randomized clinical trial. Nutr. Neurosci..

[127] Cheng Y.C., Liu J.R. (2020). Effect of *Lactobacillus rhamnosus* GG on Energy Metabolism, Leptin Resistance, and Gut Microbiota in Mice with Diet-Induced Obesity. Nutrients.

[128] Wang B., Yu H., He Y., Wen L., Gu J., Wang X., Miao X., Qiu G., Wang H. (2021). Effect of soybean insoluble dietary fiber on prevention of obesity in high-fat diet fed mice via regulation of the gut microbiota. Food Funct..

[129] Russell J.T., Zhou Y., Weinstock G.M., Bubier J.A. (2021). The Gut Microbiome and Substance Use Disorder. Front Neurosci..

[130] Dietert R.R., Dietert J.M. (2021). Microbiome First Approaches in Pain Prevention and Management. Am. J. Biomed. Sci. Res..

[131] Forouzan S., McGrew K., Kosten T.A. (2021). Drugs and bugs: Negative affect, psychostimulant use and withdrawal, and the microbiome. Am. J. Addict..

[132] O’Sullivan S.J., Schwaber J.S. (2021). Similarities in alcohol and opioid withdrawal syndromes suggest common negative reinforcement mechanisms involving the interoceptive antireward pathway. Neurosci. Biobehav. Rev..

[133] Xiao H.W., Ge C., Feng G.X., Li Y., Luo D., Dong J.L., Li H., Wang H., Cui M., Fan S.J. (2018). Gut microbiota modulates alcohol withdrawal-induced anxiety in mice. Toxicol. Lett..

[134] Zhao W., Hu Y., Li C., Li N., Zhu S., Tan X., Li M., Zhang Y., Xu Z., Ding Z. (2020). Transplantation of fecal microbiota from patients with alcoholism induces anxiety/depression behaviors and decreases brain mGluR1/PKC ε levels in mouse. Biofactors.

[135] Rodriguez-Gonzalez A., Orio L. (2020). Microbiota and Alcohol Use Disorder: Are Psychobiotics a Novel Therapeutic Strategy?. Curr. Pharm. Des..

[136] Lucerne K.E., Osman A., Meckel K.R., Kiraly D.D. (2021). Contributions of neuroimmune and gut-brain signaling to vulnerability of developing substance use disorders. Neuropharmacology.

[137] Freedman Z.G., Kane J.A., King T.S., Graziane N.M. (2021). The effect of prescribing antibiotics with opioids on the development of opioid use disorder: A national database study. J. Addict. Dis..

[138] Ezquer F., Quintanilla M.E., Moya-Flores F., Morales P., Munita J.M., Olivares B., Landskron G., Hermoso M.A., Ezquer M., Herrera-Marschitz M. (2021). Innate gut microbiota predisposes to high alcohol consumption. Addict. Biol..

[139] Carbia C., Lannoy S., Maurage P., López-Caneda E., O’Riordan K.J., Dinan T.G., Cryan J.F. (2021). A biological framework for emotional dysregulation in alcohol misuse: From gut to brain. Mol. Psychiatry.

[140] García-Cabrerizo R., Carbia C., O’ Riordan K.J., Schellekens H., Cryan J.F. (2021). Microbiota-gut-brain axis as a regulator of reward processes. J. Neurochem..

[141] Novelle M.G. (2021). Decoding the Role of Gut-Microbiome in the Food Addiction Paradigm. Int. J. Environ. Res. Public Health.

[142] Agustí A., Campillo I., Balzano T., Benítez-Páez A., López-Almela I., Romaní-Pérez M., Forteza J., Felipo V., Avena N.M., Sanz Y. (2021). Bacteroides uniformis CECT 7771 Modulates the Brain Reward Response to Reduce Binge Eating and Anxiety-Like Behavior in Rat. Mol. Neurobiol..

[143] Thomaz A.C., Iyer V., Woodward T.J., Hohmann A.G. (2021). Fecal microbiota transplantation and antibiotic treatment attenuate naloxone-precipitated opioid withdrawal in morphine-dependent mice. Exp. Neurol..

[144] Zawertailo L., Attwells S., deRuiter W.K., Le T.L., Dawson D., Selby P. (2020). Food Addiction and Tobacco Use Disorder: Common Liability and Shared Mechanisms. Nutrients.

[145] Morrow J.D., Castaldi P.J., Chase R.P., Yun J.H., Lee S., Liu Y.Y., Hersh C.P. (2021). Peripheral blood microbial signatures in current and former smokers. Sci. Rep..

[146] Prakash A., Peters B.A., Cobbs E., Beggs D., Choi H., Li H., Hayes R.B., Ahn J. (2021). Tobacco Smoking and the Fecal Microbiome in a Large, Multi-ethnic Cohort. Cancer Epidemiol. Biomark. Prev..

[147] Kosciolek T., Victor T.A., Kuplicki R., Rossi M., Estaki M., Ackermann G., Knight R., Paulus M.P., Tulsa 1000 Investigators (2021). Individuals with substance use disorders have a distinct oral microbiome pattern. Brain Behav. Immun. Health.

[148] Gopinath D., Wie C.C., Banerjee M., Thangavelu L., Kumar R.P., Nallaswamy D., Botelho M.G., Johnson N.W. (2021). Compositional profile of mucosal bacteriome of smokers and smokeless tobacco users. Clin. Oral Investig..

[149] He L., Zhou Y.X., Zhang Y., Hang B., Chang H., Schick S.F., Celniker S.E., Xia Y., Snijders A.M., Mao J.H. (2021). Thirdhand cigarette smoke leads to age-dependent and persistent alterations in the cecal microbiome of mice. MicrobiologyOpen.

[150] Kelley S.T., Liu W., Quintana P.J.E., Hoh E., Dodder N.G., Mahabee-Gittens E.M., Padilla S., Ogden S., Frenzel S., Sisk-Hackworth L. (2021). Altered microbiomes in thirdhand smoke-exposed children and their home environments. Pediatr. Res..

[151] Biedermann L., Zeitz J., Mwinyi J., Sutter-Minder E., Rehman A., Ott S.J., Steurer-Stey C., Frei A., Frei P., Scharl M. (2013). Smoking cessation induces profound changes in the composition of the intestinal microbiota in humans. PLoS ONE.

[152] Alberg A.J., Carter C.L., Carpenter M.J. (2007). Weight gain as an impediment to cigarette smoking cessation: A lingering problem in need of solutions. Prev. Med..

[153] Simpson S., Kimbrough A., Boomhower B., McLellan R., Hughes M., Shankar K., de Guglielmo G., George O. (2020). Depletion of the Microbiome Alters the Recruitment of Neuronal Ensembles of Oxycodone Intoxication and Withdrawal. eNeuro.

[154] Lewis C.B., Laflin M., Gray D.L. (2021). Exercise as Medicine for Older Women. Clin. Geriatr. Med..

[155] Nascimento M.M. (2021). Dance, aging, and neuroplasticity: An integrative review. Neurocase.

[156] Carapellotti A.M., Stevenson R., Doumas M. (2020). The efficacy of dance for improving motor impairments, non-motor symptoms, and quality of life in Parkinson’s disease: A systematic review and meta-analysis. PLoS ONE.

[157] Ruiz-Muelle A., López-Rodríguez M.M. (2019). Dance for People with Alzheimer’s Disease: A Systematic Review. Curr. Alzheimer Res..

[158] Sakellariou X.M., Papafaklis M.I., Domouzoglou E.M., Katsouras C.S., Michalis L.K., Naka K.K. (2021). Exercise-mediated adaptations in vascular function and structure: Beneficial effects in coronary artery disease. World J. Cardiol..

[159] Cominato L., Franco R., Damiani D. (2021). Adolescent obesity treatments: News, views, and evidence. Arch. Endocrinol. Metab..

[160] Richards N.C., Gouda H.N., Durham J., Rampatige R., Rodney A., Whittaker M. (2016). Disability, noncommunicable disease and health information. Bull. World Health Organ..

[161] Lee M.C., Hsu Y.J., Ho H.H., Kuo Y.W., Lin W.Y., Tsai S.Y., Chen W.L., Lin C.L., Huang C.C. (2021). Effectiveness of human-origin Lactobacillus plantarum PL-02 in improving muscle mass, exercise performance and anti-fatigue. Sci. Rep..

[162] Agosti M., Tandoi F., Morlacchi L., Bossi A. (2017). Nutritional and metabolic programming during the first thousand days of life. Pediatr. Med. Chir..

[163] Reddy S.P., Mbewu A.D. (2016). The Implications of the Developmental Origins of Health and Disease on Public Health Policy and Health Promotion in South Africa. Healthcare.

[164] Barker D.J. (1990). The fetal and infant origins of adult disease. BMJ.

[165] Hanson M., Gluckman P. (2011). Developmental origins of noncommunicable disease: Population and public health implications. Am. J. Clin. Nutr..

[166] Adair L.S. (2014). Long-term consequences of nutrition and growth in early childhood and possible preventive interventions. Nestlé Nutr. Inst. Workshop Ser..

[167] Raspini B., Porri D., De Giuseppe R., Chieppa M., Liso M., Cerbo R.M., Civardi E., Garofoli F., Monti M.C., Vacca M. (2020). Prenatal and postnatal determinants in shaping offspring’s microbiome in the first 1000 days: Study protocol and preliminary results at one month of life. Ital. J. Pediatr..

[168] Scher M.S. (2021). “The First Thousand Days” Define a Fetal/Neonatal Neurology Program. Front. Pediatr..

[169] Subcomisión DOHaD—SAP “Origen de la Salud y Enfermedad en el Curso de la Vida”—Sociedad Argentina de Pediatría (2020). Concepto de Developmental Origins of Health and Disease: El ambiente en los primeros mil días de vida y su asociación con las enfermedades no transmisibles [Developmental Origins of Health and Disease Concept: The environment in the first 1000 days of life and its association with noncommunicable diseases]. Arch. Argent. Pediatr..

[170] Moreno Villares J.M., Collado M.C., Larqué E., Leis Trabazo R., Saenz De Pipaón M., Moreno Aznar L.A. (2019). Los primeros 1000 días: Una oportunidad para reducir la carga de las enfermedades no transmisibles [The first 1000 days: An opportunity to reduce the burden of noncommunicable diseases]. Nutr. Hosp..

[171] Chilvers A. (2016). The first 1000 days. The impact of microbes on health outcomes. J. Fam. Health.

[172] Schwartzenberger S.J. (2018). Your Baby’s First 1000 Days: AAP Policy Explained.

